# Receptor Binding Drives Toxicokinetic Processes: Insights from Two Amphipod Species Exposed to Insecticides Targeting the Nicotinic Acetylcholine Receptor

**DOI:** 10.1021/acs.est.6c08872

**Published:** 2026-10-15

**Authors:** Clarissa von Au, Natsumi Maeda, Johannes Raths, Juliane Hollender

**Affiliations:** † Department of Environmental Chemistry, 28499 Swiss Federal Institute of Aquatic Science and Technology - Eawag, Dübendorf 8600, Switzerland; ‡ Institute of Biogeochemistry and Pollutant Dynamics, ETH Zürich, Zürich 8092, Switzerland

**Keywords:** Bioaccumulation, nAChR binding, neonicotinoid insecticides, aquatic invertebrates, Hyalella azteca, Gammarus pulex

## Abstract

Insecticides targeting nicotinic acetylcholine receptors (nAChRs) are used because of their high neuroactive efficacy. However, they can also exert substantial toxic pressure on nontarget aquatic invertebrates. A previous study suggested that irreversible receptor binding is the main driver of thiacloprid bioaccumulation. To determine whether the observed elimination resistance applies to other nAChR-binding insecticides and species, this study investigates five compounds from different substance classes that target nAChRs. Using the two amphipod species, *Hyalella azteca* and *Gammarus pulex*, toxicokinetic experiments were combined with assessing receptor binding *in vivo* and *in vitro*. The three N-cyanoamidines and N-nitroguanidine neonicotinoids (thiacloprid, acetamiprid, and imidacloprid) and the metabolite imidacloprid-olefin showed resistance toward elimination (i.e., over 15 days of depuration in both amphipod species). This observation could be well described by a two-compartment toxicokinetic (TK)-receptor model (>0.89 *R* ^2^) with integrated irreversible binding. In contrast, the butenolide flupyradifurone was fully eliminated, and the organic biocide spinosad was eliminated significantly but slowly, both fitted well with a one-compartment TK model (>0.71 *R* ^2^). These findings demonstrate that receptor-binding specificity and affinity are highly compound class dependent. Although no direct link between receptor binding and acute toxicity could be established, elimination resistance indicates a risk for delayed, sublethal effects that should be critically considered in regulatory risk assessment.

## Graphical abstract

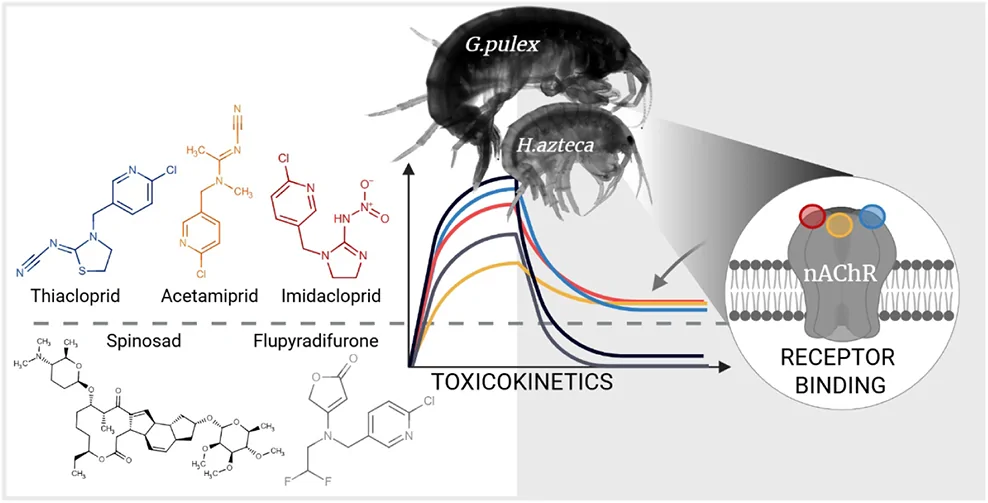

## Introduction

The widespread application of pesticides to secure and enhance agricultural production poses a global pollution risk, affecting not only terrestrial but also aquatic ecosystems.^1–3^ Among the different types of pesticides, insecticides have the most profound ecological impacts, particularly because of their high potency beyond target organisms.^4,5^ On a global scale, neonicotinoids are the most widely applied insecticide class.^6–8^ In Europe, the agricultural use of neonicotinoids has been gradually restricted, with acetamiprid (ACE) remaining the only neonicotinoid still approved in the EU and Switzerland, though its approval is currently under review.^6^ Yet, veterinary medicines can also significantly contribute to surface water pollution with neonicotinoids.^7^ Despite decreasing overall insecticide application amounts, environmental concerns are driven primarily by highly potent pyrethroids and neonicotinoids. They are frequently detected, often occur in mixtures, and exceed chronic toxicity thresholds, increasing toxicity risks to a wide range of aquatic invertebrates and pollinators.^8–10^ Neonicotinoids substantially influence the total toxic pressure on nontarget invertebrates, as constant internal concentrations in model species like *Gammarus pulex* persist, regardless of fluctuating water concentrations following, for example, agricultural runoff events.^11–14^ Accordingly, incomplete elimination of thiacloprid (THI) has been observed independently of concentration levels or dynamic exposure patterns, in both laboratory and outdoor mesocosm settings, and linked to irreversible binding to transmembrane proteins.^15,16^ Mechanistically, neonicotinoids target the nicotinic acetylcholine receptors (nAChRs) involved in neurotransmission.^17–19^ Notably, differences in receptor properties among species or tissues have been shown to influence the sensitivity of several invertebrates toward receptor-binding compounds.^20–22^ In environmental risk assessments, toxicokinetic (TK) models provide a framework to predict the time-dependent processes of uptake and elimination that drive bioaccumulation and subsequent biological effects of chemical contaminants.^23,24^ While lipid partitioning (log*K*_ow_ > 3) is established as a driver of bioaccumulation potential,^25^ additional mechanisms (e.g., protein chemical surface-binding^26^) were recognized as contributing factors, yet they have not been incorporated into standard TK models and risk assessment. A two-compartment model could better describe the slow elimination of imidacloprid (IMI) in *Hyalella azteca* ^27^ and a two-compartment receptor model was developed to describe the observed elimination resistance of THI in *G. pulex*.^15^ Whether this receptor model is applicable to other insecticides and species has not yet been tested.

Thus, this study aims to enhance our understanding of receptor binding as a contributing factor to bioaccumulation. We hypothesized that different binding modes exhibit distinct elimination-resistant profiles and that these characteristics can be captured by toxicokinetic modeling, ultimately helping to explain differences in species sensitivity. To test this hypothesis, we performed several uptake-elimination experiments as well as receptor-binding assays using two key amphipod model species the North American *H. azteca* and the European *G. pulex*. Five insecticides targeting nAChRs with three modes of action were selected (Figure 1).

**Figure 1:**
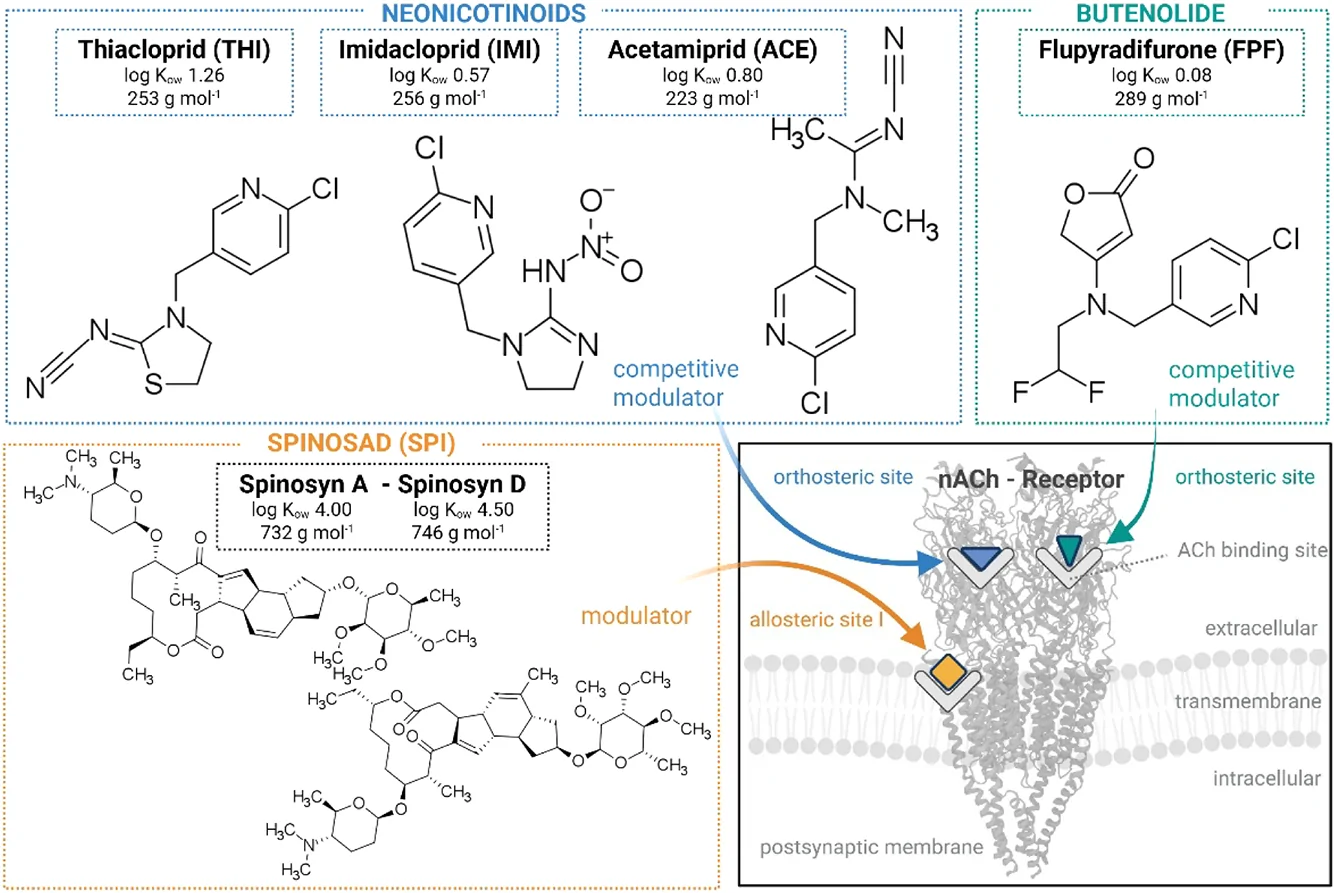
Chemical structures (derived from Chemaxon) and the chemical properties (derived from PubChem^28^) of the tested insecticides: Three neonicotinoids (i.e., THI, IMI, ACE), one butenolide (FPF), and one biopesticide (SPI) binding at different sites of an acetylcholine receptor (AChR).

The cyclic N-nitroimine and N-cyanoimine neonicotinoids IMI and THI, in addition to the acyclic N-cyanoimine neonicotinoid acetamiprid (ACE), act as competitive agonists on the nAChR.^29^ We further included the butenolide flupyradifurone (FPF) that has emerged as a potential alternative from a different chemical class and acts as a competitive modulator. All four substances are neutral and very polar (for p*K*_a_ and log*K*_ow_ details see Supporting Information section 1, SI-A-S1), suggesting low bioaccumulation potential. Additionally, the macrocyclic biocide spinosad (SPI), a nonsynthetic insecticide used in organic farming, which consists of a mixture of two analogs, spinosyn A and spinosyn D, has been selected for its allosteric activation of nAChRs at a distinct binding site not targeted by neonicotinoids.^30,31^ Spinosad is more lipophilic than the other compounds and exists predominantly in an ionized, cationic state (SI-A-S1), suggesting moderate bioaccumulation potential.^32^

## Materials and Methods

### Test Compounds

Five insecticides, thiacloprid (THI), imidacloprid (IMI), acetamiprid (ACE), flupyradifurone (FPF), and spinosad (SPI), were selected for experiments (Figure 1). Reference standards of imidacloprid-olefin (IMI-ole), 3-hydroxy-imidacloprid (3-OH-IMI), imidacloprid-urea (IMI-urea), desnitro-imidacloprid hydrochloride (IMI-desnitro), thiacloprid amide (THI-amide), and acetamiprid-N-Desmethy (ACE-N-Des) were used to screen for potential biotransformation. Thiacloprid-d4 (THI-d4), imidacloprid-d4 (IMI-d4), acetamiprid-d3 (ACE-d3) and flupyradifurone-d5 (FPF-d5) were used as isotope-labeled internal standards, while clarithromycin-d3 (CLR-d3) served as a structural surrogate internal standard for quantification (additional information in SI-A-S1).

### Test Animals

Two amphipod species, *G. pulex* and *H. azteca*, were used in the experiments (Figure SI-A1). Amphipod crustaceans are not regulated under animal experimentation legislation according to the European Directive 2010/63/EU or the Swiss Animal Welfare Act (AniWA; SR 455). Nevertheless, all organism husbandry and handling were performed in accordance with internal standard operating procedures of Eawag, with careful consideration of animal care.

Genetic specifications of the populations are provided elsewhere.^33^ *G. pulex* were collected from a reference site near Zürich, Switzerland (47.2749 °N, 8.7892 °E)^15,33,34^ between December 2023 and December 2025. Both male and female organisms were randomly selected. Juvenile or visibly infected animals with acanthocephalan parasites were excluded from experiments. The organisms were transported to the laboratory and transferred to 6 L glass aquaria filled with river water from the sampling site. During the acclimation period prior to the experiments (≥3 days), the water was progressively replaced to 100% artificial pond water (APW).^35^ The aquarium was aerated under a 12:12 h light-dark cycle in a climate chamber at 15 °C. The organisms were fed ad libitum with leaves collected at the sampling site. *H. azteca* were obtained from a laboratory culture. Adult males and females (≥3–6 months old) were randomly selected. During the acclimation period prior to the start of the experiments (≥2 days), the organisms were transferred from the culture to 6 L glass aquaria filled with Borgmann medium.^36^ The aquaria were aerated under a 12:12 h light-dark cycle in an incubator at 21 °C, and organisms were fed with milled fish food flakes (TetraMin).

Measured concentrations (SI–B) and lipid and protein content (Table SI-A2) are reported on a wet-weight [w.w.] basis but can be converted using an experimental factor of 4.8 for *G. pulex* and 4.1 for *H. azteca* (Table SI-A3). Details on protein extraction and quantification are provided under sample preparation.^37^ Across quantifications (Table SI-A2), the total protein content of *H. azteca* was 6.2 ± 0.9% (n = 11), with a membrane protein (MP) content of 0.9 ± 0.2% (n = 10). In comparison, the total protein content of *G. pulex* was 4.4 ± 0.8% (n = 15), with an MP content of 0.8 ± 0.1% (n = 10). Thus, the protein content of *H. azteca* was approximately 1.4 times that of *G. pulex*, whereas the MP content was comparable. The MP content for *G. pulex* was slightly below the value (1.0 ± 0.1%) reported by Raths et al.^15^ The gravimetrically determined lipid content^37^ for *H. azteca* was 3.3 ± 0.2%, and for *G. pulex* 1.7 ± 1.0%, with a range from 0.3% to 4.5% depending on seasonality and sex (Figure SI-A2).

## Experimental Design and End Points

Figure 2 depicts the experiments performed and the end points analyzed. Uptake-elimination experiments involved one uptake phase of 2 to 3 days, during which organisms were exposed to the individual test substance, without food. This was followed by an elimination phase ranging from 3 to 15 days, where organisms were transferred into a contaminant-free medium that consistently contained food (i.e., leaves for *G. pulex* or DECOTABs^37^ for *H. azteca*). Medium and food were renewed during the elimination phase after 3 to 6 days. In total, 19 separate toxicokinetic experiments were conducted between December 2023 and February 2026 (Table SI-A4). Exposure concentrations were kept below literature-derived sublethal levels (Table SI-A5), with mortality across the entire experimental duration ranging from 4.6% to 20.8% across experiments (Table SI-A6). The pH across experiments remained stable at 7.6 ± 0.4 while temperature was maintained at 15.0 ± 0.5 °C and 21 ± 1 °C for *G. pulex* and *H. azteca*, respectively (Table SI-A6). Details on the experimental conditions for each experiment (e.g., pH, temperature, O_2_ content, mortality) are provided in SI-B (B1–B12).

**Figure 2:**
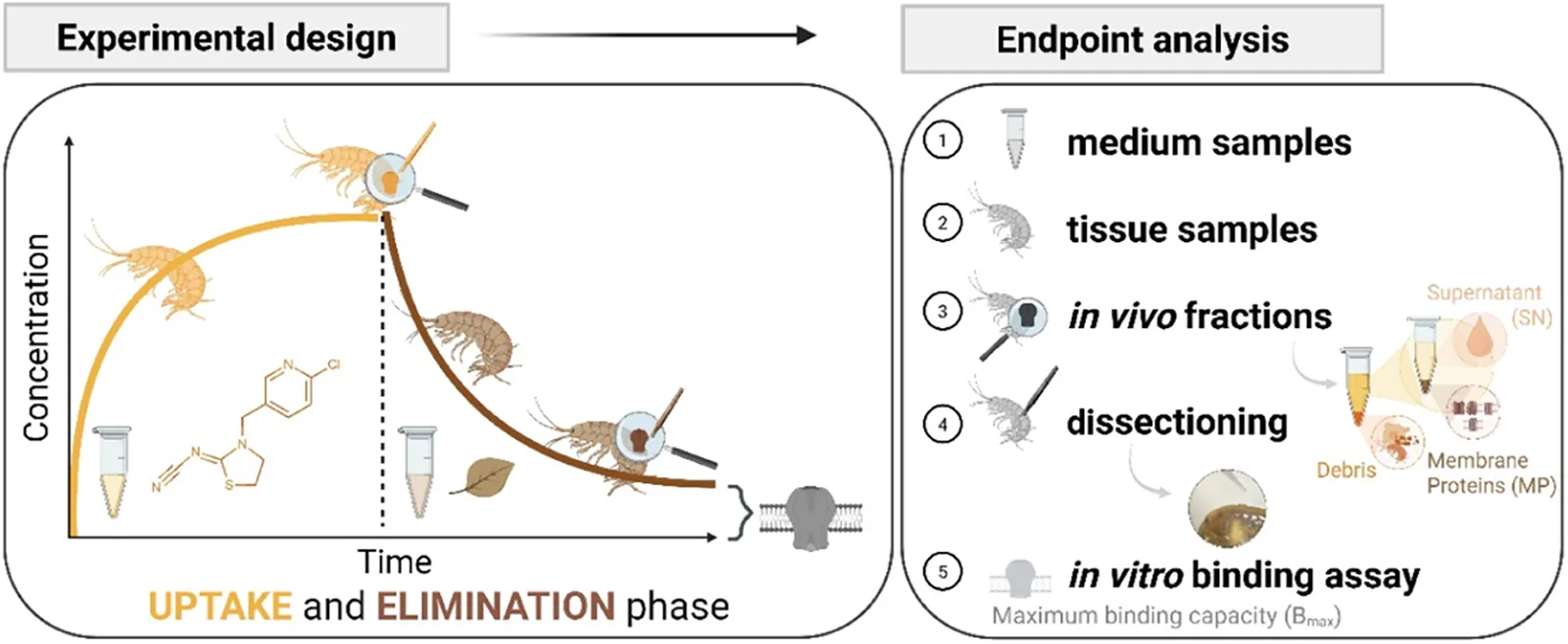
Graphical overview of the experimental design and end points analyzed. Illustrated for *G. pulex* as an example.

Medium samples were collected at the beginning and end of each exposure and elimination phase. Throughout the experiment, amphipod samples for further tissue analysis were collected in duplicate or triplicate at regular time intervals. One replicate comprised four or ten individuals for *G. pulex* and *H. azteca*, respectively. At each sampling time point, living organisms were collected, rinsed with nanopure water, dry blotted, weighed [w.w.], snap-frozen in liquid nitrogen, and stored at −20 °C until extraction.

To elucidate the spatial distribution of the contaminants in the organism, additional organism samples were collected for suborganismal investigations at the end of the uptake phase and during the elimination phase. Samples were analyzed in two ways: The organ distribution was assessed after dissection of the amphipod tissue, and the subcellular distribution was assessed by determining the *in vivo* receptor-bound fraction. For the dissection, gammarids were tranquilized on ice, their hemolymph^38^ was extracted, and the animals were further dissected^39^ into cephalon (head), intestinal system (hepatopancreas and intestine), and remaining tissue (carcass and gills) (Figure SI-A3). The dissected compartments of six gammarids were pooled. For the *in vivo* receptor-bound fraction, exposed organisms were sampled at the end of the exposure and during the elimination phase in the same manner as the tissue samples described above, except that samples were stored at −80 °C for subsequent extraction. The total body burden was fractioned into debris, the supernatant (SN), and the membrane protein-associated fraction (MP) (see next chapter). Isolation methods followed a previously adapted protocol.^15,20^

In addition to all *in vivo* experiments, an *in vitro* binding assay was conducted with isolated membrane proteins from uncontaminated control samples of *G. pulex* and *H. azteca* (SI-A-S3.3) to elucidate the binding characteristics.

### Sample Preparation and Assay Protocols

Medium samples (500 μL) and hemolymph samples were spiked with 100 μL of internal standard (ISTD mix: 250 μg L^–1^ methanol (MeOH)) and diluted with MeOH to a final volume of 1 mL.

Tissue and dissected body part samples were extracted by liquid extraction.^34^ In brief, 300 mg of 1 mm zirconia/silica beads (BioSpec Products, Inc.), 100 μL of ITSD, and 500 μL of MeOH were added before samples were homogenized using a FastPrep bead beater (two cycles of 15 s at 6 m s^–1^; MP Biomedicals). Samples were centrifuged (10,000 g, 6 min, 4 °C) and extracts were collected with syringes and filtered through 0.45 μm regenerated cellulose filters. The filters were washed with 400 μL of MeOH, and the two filtrates were combined.

Samples of the *in vivo* fractionation were homogenized using 300 mg of 1 mm zirconia/silica beads (precooled 4 °C) with a precooled tissue lyser (15 s, 6 s^–1^, 4 °C) in the first run and with the addition of dissociation medium (1 mL per sample, 4 °C) in a second homogenization run. The dissociation medium^20^ (pH = 7.0) consisted of a buffer of 20 mM sodium phosphate and 150 mM sodium chloride, as well as 0.1 mM phenylmethylsulfonyl fluoride (PMSF), 1 mM ethylenediaminetetraacetic acid (EDTA), and 0.33 mg L^–1^ pepstatin, chymostatin, and leupeptin for protease inhibition. Samples were centrifuged (30 min, 1000 g, 4 °C), and the supernatant (SN1, free and protein-bound compound) was collected with a glass pipet. After resuspension with dissociation medium (1 mL, 4 °C) and a second centrifugation step (10 min, 1000 g, 4 °C), the debris was set aside, and the supernatant (SN2) was combined with SN1 in 8 mL (adjusted with dissociation medium) ultracentrifuge vials. Samples (SN1 + SN2) were ultracentrifuged (43,000 g, 30 min, 4 °C, Ultracentrifuge CP100NX, Hitachi). Subsequently, the combined SN (free compound) was carefully removed with a glass pipet, and the pellet was resuspended (MP extract, MP bound compound).

The protein content was determined using the Pierce BCA Protein Assay Kit (Thermo Fisher Scientific) with a linear standard calibration curve (*R* ^2^ > 0.98). The total soluble protein content was quantified using a mass-balance approach. It was quantified either directly from the combined pre-ultracentrifugation fraction (SN1 + SN2 = total protein content) or after, by quantifying and summing the separated post-ultracentrifugation fractions, the combined supernatant (SN) and the MP extract (MP protein content) (SN1 + SN2 + MP = total protein content).

The *in vitro* assay used isolated membrane proteins based on the protein fractionation method described above. Pure MP extract (varying protein content between independent assays, details are provided in Table SI-A7) was added to 8 mL ultracentrifuge vials filled up to 7.5 mL with Tris–HCl buffer (10 mM, pH = 7.4) containing one of five individual compounds (i.e., IMI, THI, ACE, FPF, IMI-ole; concentration range 0.7–33 nM). The vials were incubated for 2 h at a standardized temperature of 23 °C and 100 rpm before ultracentrifugation (43,000 g, 30 min, 4 °C). Afterward, the medium was sampled, and the pellet was resuspended in 7.5 mL of Tris–HCl for cleanup and centrifuged again. Subsequently, the supernatant was removed, and the pellet was extracted for the remaining bound compounds. Each assay included a protein recovery control to correct for the MP loss during the extraction steps (Table SI-A7).

For the *in vivo* fractions and the *in vitro* assays, the SN samples were prepared as described for the medium samples. The debris and MP samples were extracted as described for the tissue samples. All samples were stored at −20 °C until chemical analysis.

### Chemical Analysis

Chemical analysis was performed using an automated online solid-phase extraction system coupled with reversed-phase liquid chromatography and a high-resolution tandem mass spectrometer (online SPE-LC-HRMS/MS; Q Exactive Plus, Thermo Fisher Scientific Inc.). Chromatographic separation was achieved with an Atlantis T3 (3 μm, 3.0 × 150 mm) column and a mobile phase gradient of nanopure water and MeOH, each with 0.1% formic acid. Specifications of the online SPE-LC-HRMS/MS settings in positive electrospray ionization mode are provided in SI-A-S4. Sample extracts (200 μL) for THI, IMI, ACE, and FPF were diluted in 20 mL of nanopure water in glass vials for the online-solid-phase extraction. For SPI, extracts were diluted in a solvent mixture composed of 8 mL MeOH and 12 mL nanopure water.

The identification and quantification of the target chemicals were based on internal standard calibration using isotope-labeled standards in Skyline (version 26.1.0.057). Injected samples were quantified by peak area ratio using the calibration curve constructed from calibration standards included in the same sample sequence. Calibrants were weighted by the inverse of their quantity in the least-squares optimization. The calibration curves for each analyte were linear over a concentration range of 0.025–250 ng mL^–1^, depending on the analyte, with *R* ^2^ > 0.99, except for spinosyn D (*R* ^2^ > 0.98). Detailed quality control parameters (i.e., relative recoveries, matrix factors and LOQs) of the online SPE-LC-HRMS/MS performance and quantification are provided in SI-B (B1–B12).

The total SPI concentration was calculated as the sum of spinosyn A and spinosyn D, each quantified according to the mean A:D ratio obtained from the calibration standard curve, relative to the internal standard CLR-d3. The ratios were determined for each measurement (i.e., G_3: A:D = 73:27, G_6: A:D = 72:28). Medium samples were quantified using a matrix-matched calibration curve (A:D = 70:30) to ensure more reliable quantification. Due to the absence of a matching isotopically labeled internal standard and the use of ratio calculations, higher quantification uncertainty is expected.

### Data Analysis and Toxicokinetic Modeling

Statistical analysis and data visualization were performed using GraphPad Prism 11.0.0 (GraphPad Software, Inc.). To maintain consistency across all data sets, including those with unequal replicate sizes and multiple time points, data were analyzed using linear mixed-effects models. Posthoc pairwise comparisons were performed using Tukey’s multiple comparisons test on the estimated marginal means (*p* < 0.05).

The toxicokinetic-receptor model built for thiacloprid in *G. pulex* ^15^ was used and extended for growth^40^ (SI-A-S5, Github). The model (Table SI-A11) simplifies the organism into a two-compartmental system with a structural compartment (S) and a receptor (MP) compartment. Consequently, the kinetic model defines the total tissue concentration *C*_*tot*_ (μmol kg^–1^) as the sum of the concentration in the structure compartment C_*S*_ (μmol kg^–1^) and the MP compartment C_*MP*_ (μmol kg_MP_^–1^) as described in eq 1. *FMS* describes the factor for the proportional correction of the membrane protein volume compared to the volume of the structure compartment and was set to *FMS* = 0.008 and 0.009 kg_MP_ kg_S_^–1^ for *G. pulex* and *H. azteca*, respectively (density assumption of 1 kg L^–1^):

1 $$C_{tot}\left(t\right)=C_{S}+C_{MP}\times FMS$$

While C_*S*_ and C_*MP*_ can be described by ordinary differential eqs 2 and 3:

dCMP(t)dt=kon×CS (t)×(Bmax−CMP(t))−koff×CMP(t)receptor−liganddissociation−g(t)×CMP(t)growthdilution(2)withBmax−CMP(t)≥0dCS(t)dt=ku×Cw(t)−ke×CS(t)−dCMP(t)dt×FMS−g(t)×CS(t)growthdilution(3)

Where *t* is the time (d), *C*_*W*_ (μM) the concentration of the exposure medium (characterized using a time-varying mode with linear interpolation between sampling points), *k*_*u*_ (L kg^–1^ d^– 1^) the uptake rate constant, *k*_*e*_ (d^–1^) the elimination rate constant, *B*_*max*_ (μmol kg^–1^) the maximal binding capacity of the MP compartment with the receptor, *k*_*on*_ (kg μmol^1^ d^–1^) the ligand–receptor complex second-order rate constant, and *k*_*off*_ (d^–1^) determines the dissociation of the complex, depending on the concentration of ligand-bound receptors. The model assumes bidirectional exchange between the structure compartment and the environment, and unidirectional exchange between the structure compartment and the MP compartment due to irreversible receptor binding (first-order rate constant *k*_*off*_ = 0), because previous work^15^ observed no elimination from the MP compartment over the course of the elimination phase (up to 8 days, *G. pulex*).^15^ The first-order growth rate constant, *g­(t)* (d^–1^), was determined on the body weight over time in all long-duration experiments (>14 days). Thus, the fixed input parameter for *H. azteca* was set to 0.059 (0.045–0.073), while no growth was observed for *G. pulex* (Details provided in SI-A-S5.2). Building the model, we assumed that the total number of receptors per unit of structural volume is constant, i.e., a fixed fraction of the structural body volume. Thus, the dilution factor scales the relationship in the same manner as for the internal concentration. All model calculations were performed in MATLAB 2021b using the Bring Your Own Model (BYOM) modeling platform (version 6.2). The Akaike Information Criterion (AIC) and *R* ^2^ were used to evaluate model performance. The parameter space explorer was used to optimize model parameters and to produce confidence intervals (CI, 95%). Different models were parametrized for each insecticide and species (SI-A-S5.3 and S5.4). Internal concentrations derived from long-duration toxicokinetic experiments (>10 days) with multiple time points were used to calibrate the model. Afterward, concentrations from shorter toxicokinetic experiments (<6 days) with two time points were used for validation. These independent data sets were intentionally deployed at a secondary concentration level to evaluate whether the calibrated parameters could capture receptor-binding patterns across different exposure concentrations (details in SI-A-S5.1). Validation predictions were evaluated with the root-mean-square error (RMSE), coefficient of variation of RMSE (CVRMSE), relative bias (% bias), or concordance correlation coefficient (CCC) (SI-A-S5.5).

Receptor binding properties from the *in vitro* assay were modeled in GraphPad Prism. The specific binding (C_*specific*_, pmol mg^–1^ membrane protein) model accounted for one-site, specific binding under equilibrium conditions, where *B*_*max*_ (pmol mg^–1^ membrane protein) is the maximal binding (maximum number of binding sites), *K*_*d*_ is the equilibrium dissociation constant (representing the binding affinity), and *C*_*Free*_ (nM) corresponds to the concentration in the medium:

4 $$C_{specific}=\frac{B_{Max}\times C_{Free}}{K_{d}+C_{Free}}$$

## Results and Discussion

### Toxicokinetics of Neonicotinoids

The toxicokinetics of the three N-cyanoamidines and N-nitroguanidine neonicotinoids showed elimination resistance and, accordingly, persistent receptor-binding was observed for both species (Figure 3).

**Figure 3:**
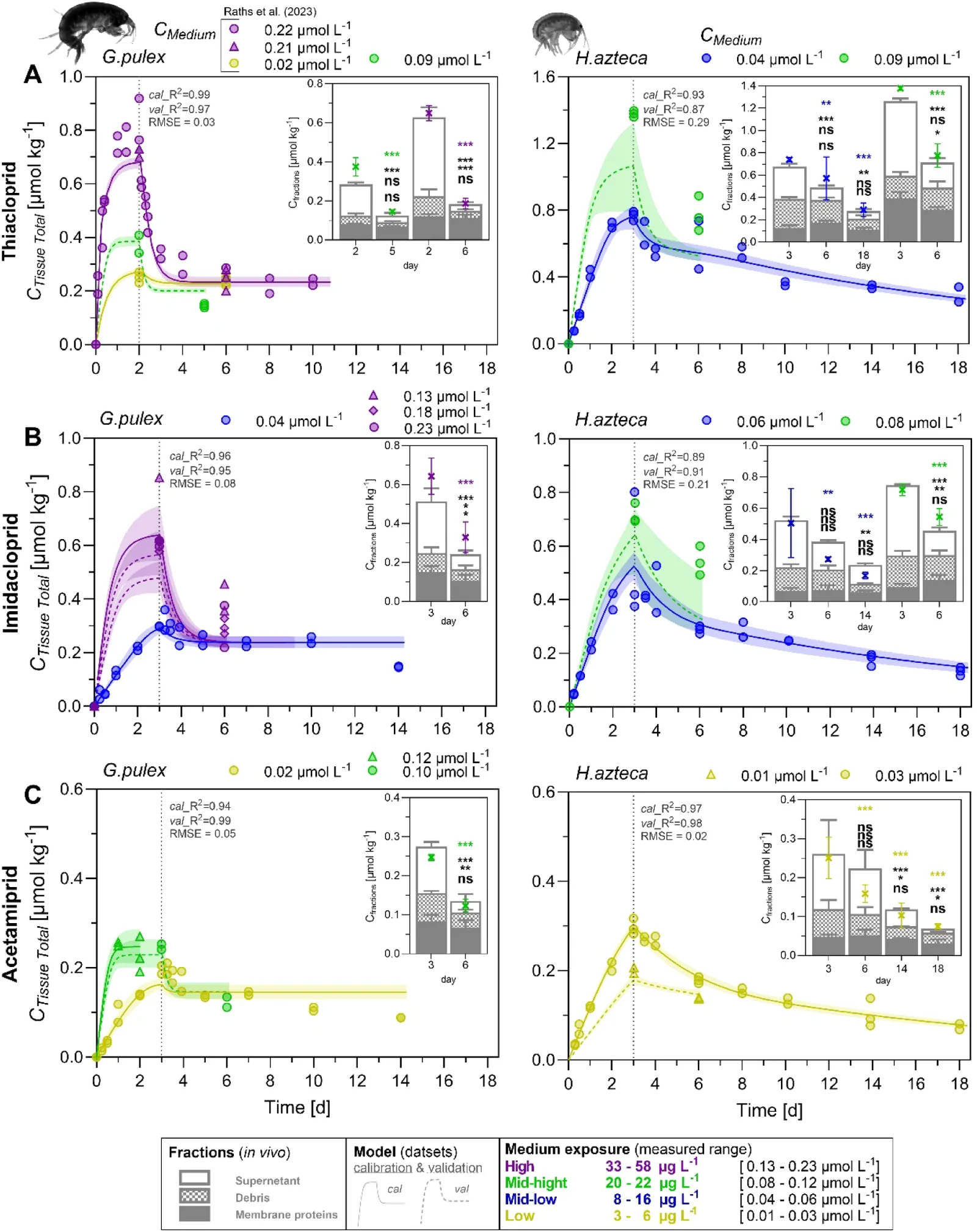
Toxicokinetics for *G. pulex* and *H. azteca* respectively for the neonicotinoids A) thiacloprid B) imidacloprid, and C) acetamiprid. Each graph shows the measured internal concentrations [μmol kg^–1^] and the model fits with the corresponding 95% confidence intervals (colored area) with the corresponding exposure media concentrations noted above each graph [μmol L^–1^]. Solid and dashed lines indicate the model fits for the calibration and validation data sets, respectively. Colors denote exposure tiers [μg L^–1^] across the uptake phase (2–3 d), separated by vertical dotted lines from the elimination phases (8–15 d). The inset in each graph depicts the corresponding concentrations [μmol kg^–1^] recovered in different *in vivo* fractions are presented as stacked bar plots. Significant decreases from the end of uptake to the end of the elimination phase are indicated by asterisks (n.s. = *p* ≥ 0.05, **= *p* < 0.01, ***= *p* < 0.001; mixed-effect analysis, Tukey’s posthoc test) for each concentration experiment individually. Note the scale differences on the *y*-axis for each compound and species. Data at 0.02, 0.21, and 0.22 μmol L^–1^ for THI (*G. pulex*) is initially published in Raths et al.^15^ Refer to Figure 1 for the molecular masses of each compound to facilitate conversion between molar units and μg L^–1^.

Looking at the kinetics in detail, we can see that internal tissue concentrations increased during the 72-h exposure phase. For both species, the relative bioconcentration potential (i.e., THI > IMI > ACE) of the tested compounds was consistent (Figure 3). All controls (*t*_0_) were free of the selected insecticides. The measured medium concentrations for the compounds remained constant (<6%) throughout the uptake phase (SI–B). Figure 3 presents the measured geometric mean concentrations (μmol L^–1^) for compound comparisons and exposure tiers (μg L^–1^) to simplify comparisons across species and exposure scales (see Figure 1 for molecular masses). Regardless of exposure concentration, all neonicotinoids in both species exhibited an elimination-resistant fraction throughout the study (14 days for *G. pulex* and 18 days for *H. azteca*). This aligns with observations for THI^15^ and IMI^41^ in *G. pulex* and IMI in *H. azteca*.^27^ This incomplete elimination is explained by membrane protein interactions, as confirmed by the *in vivo* receptor-binding fractions, thereby providing mechanistic evidence (Figure 3). Indeed, concentrations in the supernatant fraction were consistently the first to decline significantly from the uptake to the elimination phase and, followed by a more variable, yet significant, decrease in the debris fraction. In contrast, the MP-associated fraction remained constant during both phases and depicted no significant decline over multiple time points. Variance between fractions may be due to differences in extraction efficiency, though a protein recovery control was not evaluated.

To constrain the scope of elimination resistance to specific compartments, we analyzed tissue-specific concentrations (e.g., IMI *G. pulex*) (SI-A-S6.1). As presented in Figure SI-A15 IMI was present in hemolymph during the uptake phase (0.14 μmol kg^–1^) but below detectable levels during the elimination phase. This rapid clearance is expected, as it is a soluble compartment that equilibrates quickly with the surrounding medium and can be associated with the clearance observed for the supernatant fraction. A significant difference was also observed in the intestinal system, where concentrations (1.17 μmol kg^–1^) were higher than those in whole-body tissue (0.60 μmol kg^–1^) during the uptake phase, despite the animals not being fed during this period. This suggests that the intestinal tissue is a primary site for transepithelial absorption.^42,43^ However, the subsequent 84% reduction in the intestinal system concentration during the elimination phase (0.19 μmol kg^–1^) indicates that this compartment is characterized by high metabolic turnover or rapid desorption. Both structural compartments (i.e., carcass and cephalon) closely followed the significant decline from uptake to elimination observed in whole-body tissue (49%), with 51% of the burden remaining in each compartment. This localization identifies these compartments as the primary sites of elimination resistance, consistent with their presence in nerve cord and muscle tissue, including the target nAChRs.

The observed elimination resistance is best captured by a multicompartment model, which accounts for both rapid systemic clearance and slow, receptor-dependent depuration.^15^ The determined model parameters for the applied two-compartment models are presented in Table 1, and model fits are shown in Figure 3. Previous studies applied a one-compartment model and neglected remaining concentrations after 3 or 6 days of elimination. For the example of *G. pulex* exposed to IMI, at higher exposure concentrations (i.e., 86 μg L^–1^), near-complete depuration (t_95_ = 11.2 d, *k*_*e*_ = 0.27 d^–1^)^44^ was predicted, which overestimated the initial clearance and failed to account for the remaining fraction observed at day 14. Whereas at similar concentrations (i.e., 11 μg L^–1^), a slower depuration rate was predicted (*k*_*e*_= 0.12 d^–1^).^45^ Ultimately, the two-compartment model reflects much better the biphasic elimination characteristics,^15,27^ which characterized a substantially faster initial depuration rate (*k*_*e*_ = 1.7 d^–1^). To account for slow apparent elimination after the rapid initial clearance during the depuration phase, the receptor model for *H. azteca* was extended with growth dilution (eqs 2 and 3). This was supported by food availability in the elimination phase and consistent growth rates observed in our laboratory culture. In contrast, no significant growth was observed in the field-collected organisms *G. pulex*, likely due to the heterogeneous age/size variability (detailed information in SI-A-S5.2). To definitely conclude that our temporal resolution captures irreversibility, we supplemented our study with a long-term experiment (*G. pulex*, with THI) extending up to 60 days of elimination (SI-A-S6.2), which demonstrates negligible elimination in total tissue concentrations over a long elimination time (Figure SI-A16). Nonetheless, we tested a modeled reversible binding (*k*_*off*_ > 0, Table SI-A12), but it was insufficient to explain the prolonged slow elimination we observed in Figure 3. Consequently, the model supports irreversible receptor binding (*k*_*off*_ = 0) and highlights that physiological factors (e.g., growth), rather than dissociation, can govern the observed concentration decline (SI-A-S5.3). Furthermore, considering growth dilution allowed a more consistent parameter estimation for *k*_*e*_ than a previous approach^16^ without growth. Having fixed the dissociation rate, we further refined the parametrization by addressing the association process (*k*_*on*_). Model applications across all compounds showed that the association process toward the receptor is quasi-instantaneous relative to the uptake rate, within the given temporal resolution. Thus, the parameter was set to an established default value (200 kg μmol^1^ d^–1^)^15^ (Table 1), thereby improving computational efficiency. Model fits are in agreement with the measured data (*R* ^2^ = 0.89–0.99), and the profile likelihoods of *k*_*u*_, *k*_*e*_, and *B*_*max*_ were well-defined. The two-compartment model captured the toxicokinetic trend in the validation data sets (*R* ^2^ = 0.87–0.99). However, validation predictions exhibit a systematic underprediction (% bias of −6% to −36%), indicating deviations in absolute accuracy when models calibrated at a single concentration are tested against independent data sets at a secondary concentration. Details are provided in Figure 3, Table SI-A14, and Figures SI-A6-A11. Uptake rate constants for *H. azteca* and *G. pulex* are within the same order of magnitude (Table 1). Despite its larger surface-to-volume ratio and elevated experimental temperature (21 °C vs 15 °C), *H. azteca* exhibits consistently slower elimination rates. Temperature-correcting the kinetic parameters using the Arrhenius temperature (*T*_*A*_ *=* 8050 calculated from estimates^16^), confirms physiological factors govern these differences (SI-A-S5.6). Consequently, *H. azteca* displays higher kinetic bioconcentration factors (*BCF*_*kin*_) ranging from 5.6 to 21.8 L kg^–1^ across compounds compared to *G. pulex* (0.9–1.7 L kg^–1^) (Table SI-A18). Because *B*_*max*_ is temperature-independent,^16^ modeled values can be directly compared between species, depicting similarity for IMI and ACE (Table 1), while for THI, *H. azteca* exhibits significantly higher values than *G. pulex* that align with the higher concentrations observed in the *in vivo* receptor-associated fraction (inserts Figure 3; Figure SI-A17).

**Table 1:** Calibrated Best-Fit Model Parameters Obtained for Each Compound and Species^a^

		*k*_*u*_ Uptake rate constant	*k*_*e*_ Elimination rate constant	*B*_*max*_ Maximum binding capacity	*k*_*on*_ Association rate constant
		Best fit (95% Cl)	Best fit (95% Cl)	Best fit (95% Cl)	Best fit (95% Cl)
Compounds	Species	[L kg_Structure_^–1^ d^–1^]	[d^–1^]	[μmol kg_MP_^–1^]	[kg_Structure_ μmol^–1^ d^–1^]
Two-compartment model					
THI	*G. pulex* ^b^	10.6 (8.5– 13.6)	5.2 (4.2– 6.7)	25.0 (22.7– 29.4)	200 (37.6–200)
	*H. azteca*	9.7 (8.4– 11.3)	1.7 (0.8– 5.2)	63.4 (55.8– 71.4)	fixed to 200
IMI	*G. pulex*	3.0 (2.6– 3.6)	1.7 (1.2– 2.5)	29.8 (27.1– 32.5)	fixed to 200
	*H. azteca*	4.0 (3.3– 4.8)	0.6 (0.2– 1.8)	33.1 (21.6– 41.5)	fixed to 200
ACE	*G. pulex*	4.3 (3.3– 6.0)	5.0 (3.0– 10.8)	18.2 (16.2– 20.7)	fixed to 200
	*H. azteca*	4.9 (4.5– 5.3)	0.2 (0.1–0.4)	14.2 (10.0–17.6)	fixed to 200
One-compartment model					
FPF	*G. pulex*	1.7 (1.3– 2.2)	0.6 (0.4– 0.8)	omitted	
	*H. azteca*	0.8 (0.5– 1.2)	0.4 (0.2– 0.6)	omitted	
SPI	*G. pulex*	31.8 (27.7–36.5)	0.3 (0.3–0.4)	omitted	

a Fits correspond to the fitted graphs in SI-A-S5.3 and S5.4 (Figure SI-A6-A14). R^2^ ranged between 0.71 and 0.99 for calibration and between 0.82 and 0.99 for the validation datasets, except for *H. azteca* and FPF where validation failed (R^2^ = −0.21) (Table SI-A14).

b Model parameters from Raths et al.^15^

### Toxicokinetics—Elimination of Other nAChR Binding Insecticides

Compared to the tested neonicotinoids, the butenolide FPF showed lower, and the biocide SPI higher bioconcentration potential, but unlike neonicotinoids, they were eliminated significantly, with no persistent accumulation in the membrane-bound fraction during elimination (Figure 4).

**Figure 4:**
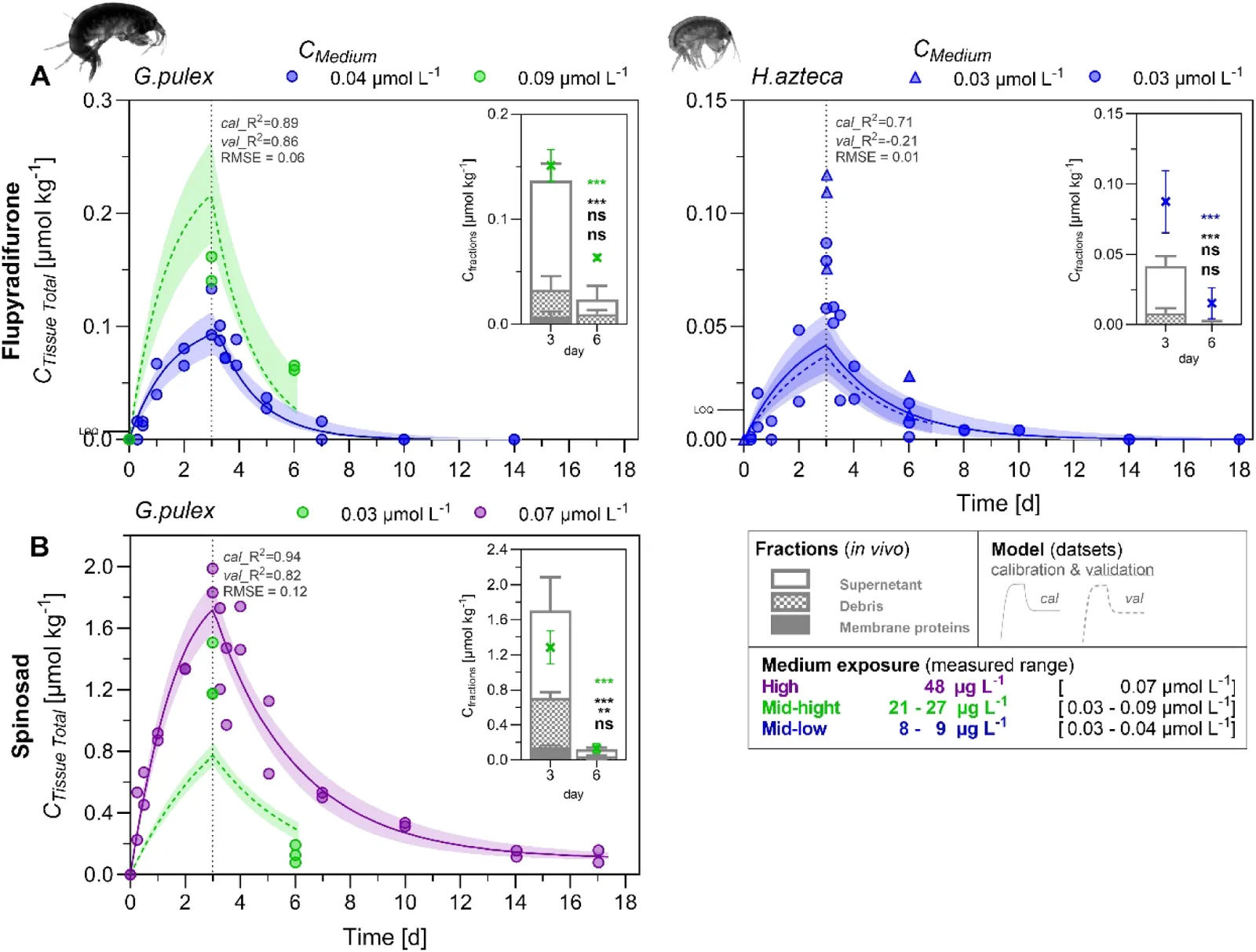
Toxicokinetics for *G. pulex* and *H. azteca* respectively for A) the butenolide flupyradifurone and B) the biocide spinosad. Each graph shows the measured internal concentrations [μmol kg^–1^] and the model fits with the corresponding 95% prediction intervals (colored area) with the corresponding exposure media concentrations noted above each graph [μmol L^–1^]. Solid and dashed lines indicate the model fits for the calibration and validation data sets, respectively. Colors denote exposure tiers [μg L^–1^] across the uptake (3 d) and elimination (11–15 d) phases, separated by vertical dotted lines. The inset in each graph depicts the corresponding concentrations [μmol kg^–1^] recovered in different fractions of the *in vivo* binding assay presented as stacked bar plots. Significant decreases from the end of uptake to the elimination phase are indicated by asterisks (n.s. = *p* ≥ 0.05, ** = *p* < 0.01, *** = *p* < 0.001; mixed-effect analysis, Tukey’s posthoc test) for each concentration experiment tested individually. Note the scale differences on the *y*-axis for each compound and species. Refer to Figure 1 for the molecular masses of each compound to facilitate conversion between molar units and μg L^–1^.

The measured FPF exposure concentrations remained constant (<8.5%) throughout the uptake phase. For SPI, the 25 μg L^–1^ exposure level remained stable, whereas the 50 μg L^–1^ treatment showed a 37% reduction after 72 h, likely due to photolytic and hydrolytic degradation reported.^46^ Thus, the geometric mean over time is reported. The highly hydrophilic compound FPF had low uptake rate constants (Table 1) and a low bioaccumulation potential with the kinetic *BCF*_*kin*_ ranging from 2.1 L kg^–1^ (*G. pulex* *)* to 2.8 L kg^–1^ (*H. azteca* *)* (Table SI-A18). For SPI, hydrophobic partitioning appears to be the primary driver for a higher uptake rate (Table 1) with a higher kinetic *BCF*_*kin*_ of 100 L kg^–1^, *G. pulex* (Table SI-A18) compared to the other compounds. To our knowledge, no other toxicokinetic studies have been reported testing SPI in aquatic invertebrates.

Given the elimination rates (Table 1) FPF fully eliminated (*t*_*95*_) within 5.0 and 8.1 days of elimination for *G. pulex* and *H. azteca*, respectively. The majority of SPI after uptake (1.91 μmol kg^–1^) was eliminated within 9.4 days (*t*_*95*_), as tested for *G. pulex* (*k*_e_ = 0.32 d^–1^), yet a small amount (0.12 μmol kg^–1^) remains at day 17. SPI was tested exclusively in *G. pulex*, since neonicotinoid data showed high cross-species consistency. Observed elimination was supported by target-site interactions identified in the *in vivo* receptor-binding assay (Figure 4). For both compounds, concentrations in the MP-associated fraction could be detected for *G. pulex* during the uptake phase, but they decreased by 95% (SPI) to 100% (FPF) during the elimination phase. For *H. aztec*a, concentrations in the MP fraction in the elimination phase fall below LOQ. This reduction confirms a reversible binding interaction (*k*_*off*_ > 0) as reported for FPF with insect nAChRs.^47^ The observed minor retention of SPI likely represents nonspecific partitioning into lipid-rich compartments (log*K*_ow_ > 4.0, un-ionized fraction) rather than receptor binding. Potential metabolite formation could have occurred yet was not explicitly screened for in this study. A comparison of one- and two-compartment model structures showed that a simplified one-compartment model explained the data equally well (ΔAIC = 4) and was thus applied (model comparison provided in Table SI-A13). The profile likelihoods of *k*_*u*_ and *k*_*e*_ were well-defined. Model fits are in agreement with the measured data for the calibration (*R* ^*2*^ = 0.71–0.94), while validation performance of the one-compartment model is highly variable (*R* ^*2*^ = −0.21–0.86) and shows systematic underprediction (% bias of −10% to −61%). Details are provided in Figure 4, Table SI-A14, and Figure SI-A12-A14. Notably, for FPF, *H. azteca* (Figure 4A, *H. azteca* and Figure SI-A14), the model fails during validation, which can be attributed to both data sets sharing a narrow range of exposure concentration. Consequently, biological variability and analytical proximity to the LOQ result in overlapping confidence intervals, illustrating the model’s predictive boundaries, while nonetheless capturing the overall kinetic trends.

### Biotransformation

Samples were screened for six known transformation products of the tested insecticides (Table SI-A1). ACE-*N*-desmethyl was identified in *G. pulex* following ACE exposure, while IMI-ole was detected in both species following IMI exposure (SI-A-S6.5). ACE-N-desmethyl was primarily detected during the uptake phase in quantifiable amounts between day 2 and 3, amounting to 2–3% of ACE by day 3. The short-term detection and the presence in the debris and supernatant fraction indicate that the metabolite is being rapidly eliminated or further transformed (Figure SI-A18). These findings extend the known N-demethylation pathways in *G. pulex* ^48^ by newly identifying ACE-N-desmethyl as a specific metabolite of acetamiprid in amphipods. In contrast to the rapid disappearance of ACE-N-desmethyl, IMI-ole remained detectable during the elimination phase, demonstrating prolonged formation (details in SI-A-S6.5). Indeed, in *G. pulex*, the IMI-ole fraction increased from 1.3% to 2.9% between the uptake and elimination phases, while in *H. azteca*, it appeared only during elimination, ranging from 5.0% to 8.1%. While IMI-ole has been previously reported in *G. pulex* at slightly higher formation ratios (4.5–7.9%)^45^ it had not been reported in earlier studies with *H. azteca*.^27^ Notably, for *G. pulex* IMI-ole was detected in the debris and MP-associated fraction, but not in the supernatant, nor the hemolymph (Figure SI-A15) with a significant increase in the MP fraction during elimination (Figure 5). This localization in specific tissue compartments (target site) provides evidence for the reported slow elimination (0 d^–1^)^45^ in *G. pulex* once formed as a metabolite and the high affinity and efficacy to the nAChR in bees,^49^ reinforcing evidence of elimination resistance similar to the parent compound. Despite the irreversible receptor-binding of IMI-ole, both metabolites were excluded from the TK model due to their minor contribution to the elimination kinetics, which fell below 10% of the total body burden across all measured time points and within the standard deviations of the parent compounds (SI–B, Figure SI-A18). A similar metabolite for thiacloprid (THI-ole) has been reported in mice,^50^ but could not be found in fish^51^ or gammarids^16^ (SI-A-S1).

**Figure 5:**
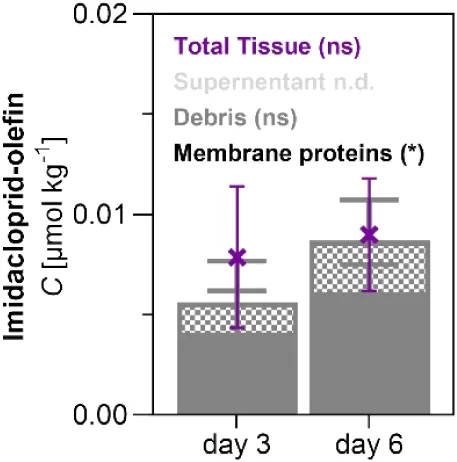
Concentrations of imidacloprid-olefin in *G. pulex* recovered in total tissue and in the different fractions of the *in vivo* binding assay presented as stacked bar plots. Significant changes from the end of uptake to the elimination phase are indicated by asterisks (* = *p* < 0.05, mixed-effect analysis, Tukey’s posthoc test).

### Binding Characteristics

To elucidate the functional differences between the orthosteric binding compounds and validate the physiological relevance of our *in vivo* observations, we conducted *in vitro* receptor-binding assays to quantify receptor binding site density (*B*_*max*_) and ligand affinity (*K*_*d*_) at concentrations corresponding to a range of 0.2 to 8 μg L^–1^ (0.7–33 nM) (Figure 6). Saturation curves of the specific binding model provided parameter estimates and overall good fits (R^2^ = 0.59–0.99, except FPF R^2^ = 0.09) (Table SI-A18). However, the high ligand-to-protein ratio precluded the differentiation of specific affinity (*K*_*d*_) differences between compounds, a technical constraint that is evaluated in further detail in SI-A-S6.6. Nonetheless, data imply that at environmentally relevant concentrations, accumulated neonicotinoids are rapidly transferred to membrane proteins (as indicated by *K*_*d*_ and *K*_*on*_), suggesting that receptors will become occupied irrespective of the concentration. Ultimately, further data analysis focused on the maximal binding capacity. THI displayed the highest binding capacity in both species, while IMI and ACE showed lower *B*_*max*_ values (Figure 6). IMI and ACE binding profiles were nearly overlapping, indicating a shared or similar receptor interaction. In contrast, *H. azteca* showed a more distinct separation, with IMI achieving a higher specific binding capacity than ACE. FPF exhibited the lowest *B*_*max*_ values overall and was detectable only for *G. pulex*, indicating a lower accessibility to the target sites among insecticides. Notably, the hierarchical binding capacity of the parent compounds (i.e., THI > IMI > ACE ≫ FPF) remains consistent, despite variations in absolute *B*_*max*_ values among species (i.e., factor 1.1 THI; 1.9 IMI; 1.6 ACE for *H. azteca*), most likely due to species-specific receptor diversities.^20^ Absolute *B*_*max*_ variations were not only observed between the two species but also between distinct *G. pulex* populations, as shown by the supplementary comparative analysis of *G. pulex* collected from Wageningen (Netherlands), though the hierarchical binding capacity remained consistent (Figure SI-A19). Further, in addition to known species-specific metabolic capacities,^52^ results highlight that target-site properties also differ, as the most substantial disparity in binding capacity was observed for the metabolite IMI-ole (8.2-fold difference) (Figure 6). For FPF, we observed a 6 to 15-fold lower specific binding capacity than for the tested neonicotinoids, suggesting restricted receptor occupancy or subtype selectivity. Similarly, a 6-fold lower affinity than the neonicotinoid IMI has been reported in bees.^53,54^

**Figure 6:**
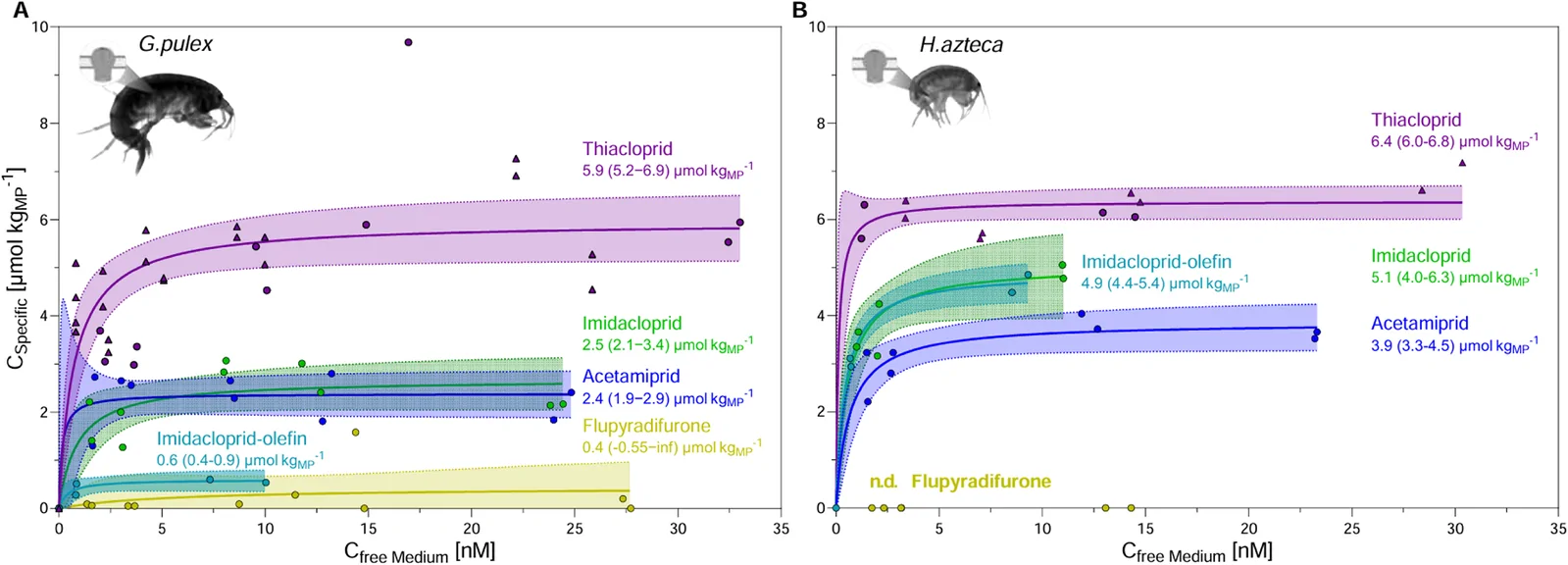
Saturation curve of the *in vitro* receptor binding assay using membrane proteins from A) *G. pulex* and B) *H. azteca*. The nonlinear regression of the one-site, specific-binding model and the 95% CIs are presented as a line and shaded area, respectively. Symbols represent measured data, while the colors represent the different compounds. For THI, two independent assays, represented by triangles and dots were performed. Data for THI (triangle, *G. pulex*) were initially published in Raths et al.^15^ Refer to Figure 1 for the molecular masses of each compound to facilitate conversion between molar units and μg L^–1^.

### Receptor Binding and Its Implications for Toxicodynamics

*In vitro B*_*max*_ values extrapolated to whole-organism concentrations (*C_B*_*max*_) resulted in a concentration range between 0.09 and 0.24 μmol kg^–1^ for THI, IMI, and ACE (Table SI-A20). These estimates capture the overall magnitude of the observed *in vivo* mean concentration range of 0.04–0.33 μmol kg^–1^ in the persistent MP fraction (inserts Table 1, Figure SI-A17) and should be interpreted as scale approximations (i.e., SI-A-S6.6 regarding methodological uncertainties). Because *B*_*max*_ is independent of temperature,^16^ *in vitro* results remain applicable when linking findings to different *in vivo* temperature conditions across species. While we saw a clear distinction in binding capacities among chemicals, we could not link observed receptor binding to species sensitivity i.e., acute lethality. No correlation between *B*_*max*_ values and toxicodynamic end points could be observed, as the compound with the highest *B*_*max*_ (i.e., THI) does not exhibit the highest lethal toxicity in either species. Based on reported lethal concentrations (Table SI-A5), the relative toxicity profiles vary by species (*G. pulex:* ACE ≫ IMI > THI^55^ and *H. azteca* ACE ≫ FPF > THI ≫ IMI^56^) and compounds (*G. pulex* > *H. azteca* for IMI sensitivity, but *H. azteca* > *G. pulex* for FPF sensitivity).^57^ Across diverse species, a higher lethality-derived hazard was derived for IMI than THI,^58^ while binding profiles showed a reverse trend, with 1.3- to 2.4-fold higher binding capacity for THI than IMI. Thus, knowing that ACE acts as a full agonist, whereas IMI and THI function as partial agonists, underlines that toxicity is governed by efficacy (receptor activation).^29,59^ Accordingly, binding affinity is not the primary determinant of toxicity, and ecotoxicological sensitivity differences are governed by other drivers, such as metabolic detoxification, rather than target-site occupancy alone.^20,43,60^ In this regard, higher affinity of IMI-ole to nAChR than its parent compound has been reported^49,61^ and we demonstrated persistent binding of IMI-ole associated with MPs. Furthermore, while compounds with reversible binding kinetics may suggest a lower risk to amphipods due to faster clearance from the target site, FPF acute toxicity for *H. azteca* is higher than that of IMI and THI. However, reversible binding may explain why FPF toxicity did not increase over time, unlike observed for IMI.^57^ *F*urther investigations at the molecular level are required to elucidate, for example, how subunit sensitivities or receptor cellular types^62^ influence the overall efficacy of these compounds. *S*ubunit combinations among the tested amphipods remain unknown and can only be inferred from studies on *Drosophila melanogaster*.^21,63,64^ Other mitigating factors, such as the presence of spatial barriers^65^ or the possibility of an off-target receptor, such as the potential presence of soluble homopentameric acetylcholine binding proteins (AChBPs) that could mitigate toxicity, have been suggested and observed in, for example, earthworm species.^22,43^ While AChBPs lack transmembrane domains,^66^ our *in vivo* results with SPI confirm that the extracted membrane-bound fraction contains the transmembrane region, where SPI is known to modulate the receptor at an allosteric site.

### Environmental Implications

We hypothesized that differential nAChR binding modes drive distinct bioaccumulation kinetics, and we indeed established a clear link between elimination resistance and persistent receptor binding for neonicotinoids. We demonstrated that accurate characterization of receptor-binding kinetics requires a two-compartment TK model, even though the underlying model assumptions may still simplify the intricate dynamics of receptor association and dissociation. The application of kinetic BCF values yields results below the regulatory threshold (i.e., BCF 2000; OECD 321) at the tested concentration range (Table SI-A18). However, the noneliminating fraction is not considered. Under steady-state, the noneliminating fraction would lead to a disproportionately larger relative contribution to the overall accumulation at lower exposure concentrations (SI-A-S6.3). Thus, the regulatory threshold would be exceeded at water concentrations in the ng L^–1^ range (i.e., ≤ 10–81 ng L^–1^ depending on the compound; Table SI-A18), which are, according to monitoring studies,^2,13,67–69^ environmentally relevant. Ultimately, our findings emphasize that target-site receptor binding is a crucial driver of bioaccumulation, highlighting the need to prioritize elimination (resistance) and giving greater priority to protein-binding assessments^26,70^ in the regulatory risk assessment of chemicals. Importantly, while toxicokinetics provide necessary evidence of exposure, they did not directly translate to toxicodynamic effects.^43^ Therefore, risk assessments must expand beyond acute lethality (e.g., behavioral end points), particularly for compounds where binding kinetics suggest a sublethal, chronic burden and time-dependent toxicity.^71,72^ Consequently, further development and integration of TKTD frameworks are required to improve the predictive power in risk assessments.

## Supplementary Material





## Data Availability

Models are shared on Github. Any questions or raw data can be available from authors on request.

## Notes

Additional experimental details, materials, and methods as well as results are provided in SI-A (PDF)
The measurement results can be found in SI-B (XLSX)

## Abbreviations

ACE: acetamiprid
Ach: acetylcholine
AChBP: acetylcholine binding protein
APW: artificial pond water
BCF: bioconcentration factor
FPF: flupyradifurone
IMI: imidacloprid
SPI: spinosad
THI: thiacloprid
TD: toxicodynamic
TK: toxicokinetic
TK–TD: toxicokinetic–toxicodynamic
MP: membrane protein
nAChR: nicotinic acetylcholine receptor
