# Supplementary material for: Receptor Binding Drives Toxicokinetic Processes: Insights from Two Amphipod Species Exposed to Insecticides Targeting the Nicotinic Acetylcholine Receptor
Source: Environ Sci Technol. 2026 Oct 15;60(42):29943–54. doi: 10.1021/acs.est.6c08872 (PMC13718619; doi:10.1021/acs.est.6c08872)
Supplement: Supplementary file 1 [file es6c08872_si_001.pdf]

Supporting Information (SI-A)

Receptor Binding Drives Toxicokinetic Processes: Insights from Two Amphipod Species Exposed to Insecticides Targeting the Nicotinic Acetylcholine Receptor

Clarissa von Au[1,2], Natsumi Maeda[1,2], Johannes Raths[1,2], and Juliane Hollender[1,2,*]

[1] Department of Environmental Chemistry, Swiss Federal Institute of Aquatic Science and Technology - Eawag, 8600 Dübendorf, Switzerland

[2] Institute of Biogeochemistry and Pollutant Dynamics, ETH Zürich, 8092 Zürich, Switzerland

[*]**Corresponding author:** juliane.hollender@eawag.ch

Contents

SI-A-S1 Chemicals **1**

SI-A-S2 Test Animals **2**

SI-A-S3 Experiments and Endpoints **5**
SI-A-S3.1 Toxicity and Mortality . . . 5
SI-A-S3.2 Dissection method . . . 6
SI-A-S3.3 *In vitro* assay . . . 7

SI-A-S4 Chemical measurement **8**

SI-A-S5 Toxicokinetic model **10**
SI-A-S5.1 Model parameters . . . 10
SI-A-S5.2 Growth considerations . . . 11
SI-A-S5.3 Two-compartment model . . . 13
SI-A-S5.4 One compartment model . . . 20
SI-A-S5.5 Model evaluation . . . 24
SI-A-S5.6 Temperature considerations . . . 25

SI-A-S6 Supplementary results **26**
SI-A-S6.1 Dissection . . . 26
SI-A-S6.2 Long-term elimination experiment (60 days) . . . 27
SI-A-S6.3 Bioconcentration factor (BCF) . . . 28
SI-A-S6.4 *In vivo* binding . . . 30
SI-A-S6.5 Biotransformation . . . 31
SI-A-S6.6 *In vitro* binding . . . 32

References **34**

SI-A-S1 Chemicals

All parent compounds and all transformation products (TPs) had a purity of >98%, and >96.8%, respectively. The deuterated analogs as internal standards (ITSDs) had purities >98%, except for FPF-d5 and CLR-d3 (>95%).
Imidacloprid (IMI), thiacloprid (THI), and acetamiprid (ACE) are neonicotinoids. Flupyradifurone (FFP) is a butenolide, and spinosad (SPI) is a biopesticide. The compounds IMI, THI, and FPF are reported as having no dissociation and for ACE the pK_a is reported as 0.7.[1] They remain in their neutral form and exist almost exclusively (>99.9%) as neutral species under test conditions (pH 7-8; SI-B). The pK_a for SPI is reported with 8.0 (mean of protonated spinosyn A and D).[2] Given the test conditions (i.e., SI-B3, SI-B6: pH 7.3 ± 0.3), it is predominantly in its ionized form (~ 72–91%).

Table SI-A1: Physico-chemical properties of the tested compounds derived from PubChem and *Regulations.gov[3] for the parent compounds, while the metabolites were calculated with Chemaxon.

Compound	Abbr.	CAS	Note	Molecular formula	Molecular weight [g mol^{-1}]	$\log K_{ow}$
Thiacloprid	THI	111988-49-9	Parent	$C_{10}H_9ClN_4S$	252.72	1.26[a]
Imidacloprid	IMI	138261-41-3	Parent	$C_9H_{10}ClN_5O_2$	255.66	0.57[b]
Acetamiprid	ACE	135410-20-7	Parent	$C_{10}H_{11}ClN_4$	222.67	0.80[c]
Flupyradifurone	FPF	951659-40-8	Parent	$C_{12}H_{11}ClF_2N_2O_2$	288.68	0.08*
Spinosad	SPI	168316-95-8	Parent	$C_{41}H_{65}NO_{10}$		
Spinosyn A		131929-60-7		$C_{41}H_{65}NO_{10}$	732.00	4.0[d]
Spinosyn D		131929-63-0		$C_{42}H_{67}NO_{10}$	746.00	4.5[d]
Thiacloprid-d4	THI-d4	1793071-39-2	ITSD	$C_{10}H_5D_4ClN_4S$	256.75	–
Imidacloprid-d4	IMI-d4	1015855-75-0	ITSD	$C_9H_6D_4ClN_5O_2$	259.68	–
Acetamiprid-d3	ACE-d3	1353869-35-8	ITSD	$C_{10}H_8D_3ClN_4$	225.69	–
Flupyradifurone-d5	FPF-d5	591659-40-8	ITSD	$C_{12}H_6D_5ClF_2N_2O_2$	293.71	–
Clarithromycin-d3*	CLR-d3	959119-22-3	ITSD	$C_{38}H_{66}D_3NO_{13}$	751.98	–
Imidacloprid-olefin	IMI-ole	115086-54-9	TP	$C_9H_8ClN_5O_2$	253.64	1.84
3-hydroxy-imidacloprid	3-OH-IMI	380912-09-4	TP	$C_9H_{10}ClN_5O_3$	271.66	1.80
Imidacloprid-urea	IMI-urea	120868-66-8	TP	$C_9H_{12}ClN_3O_2$	211.65	0.61
Desnitro-imidacloprid hydrochloride	IMI-desnitro	127202-53-3	TP	$C_9H_{11}ClN_4$	247.12	1.56
Thiacloprid amide	THI-amide	676228-91-4	TP	$C_9H_{10}ClN_3O_2S$	270.74	1.82
Acetamiprid-N-desmethyl	ACE-N-Des	190604-92-3	TP	$C_9H_9ClN_4$	208.65	1.29

[a] 20 °C; [b] 22 °C; [c] 25 °C; [d] at pH 7.

Note:
*Clarithromycin-d_3 was chosen as a surrogate internal standard due to its structural similarity involving a macrocyclic lactone core and its close chromatographic elution relative to spinosyn A and D.

For Thiacloprid-olefin, no analytical standard was available, and therefore it was excluded from our list. Furthermore, it was not identified in gammarids during HRMS screening[4] at 5 mg L^{-1},[5] which is 100 times higher than the highest concentration tested in this study ($50\ \mu$g L^{-1}).

SI-A-S2 Test Animals

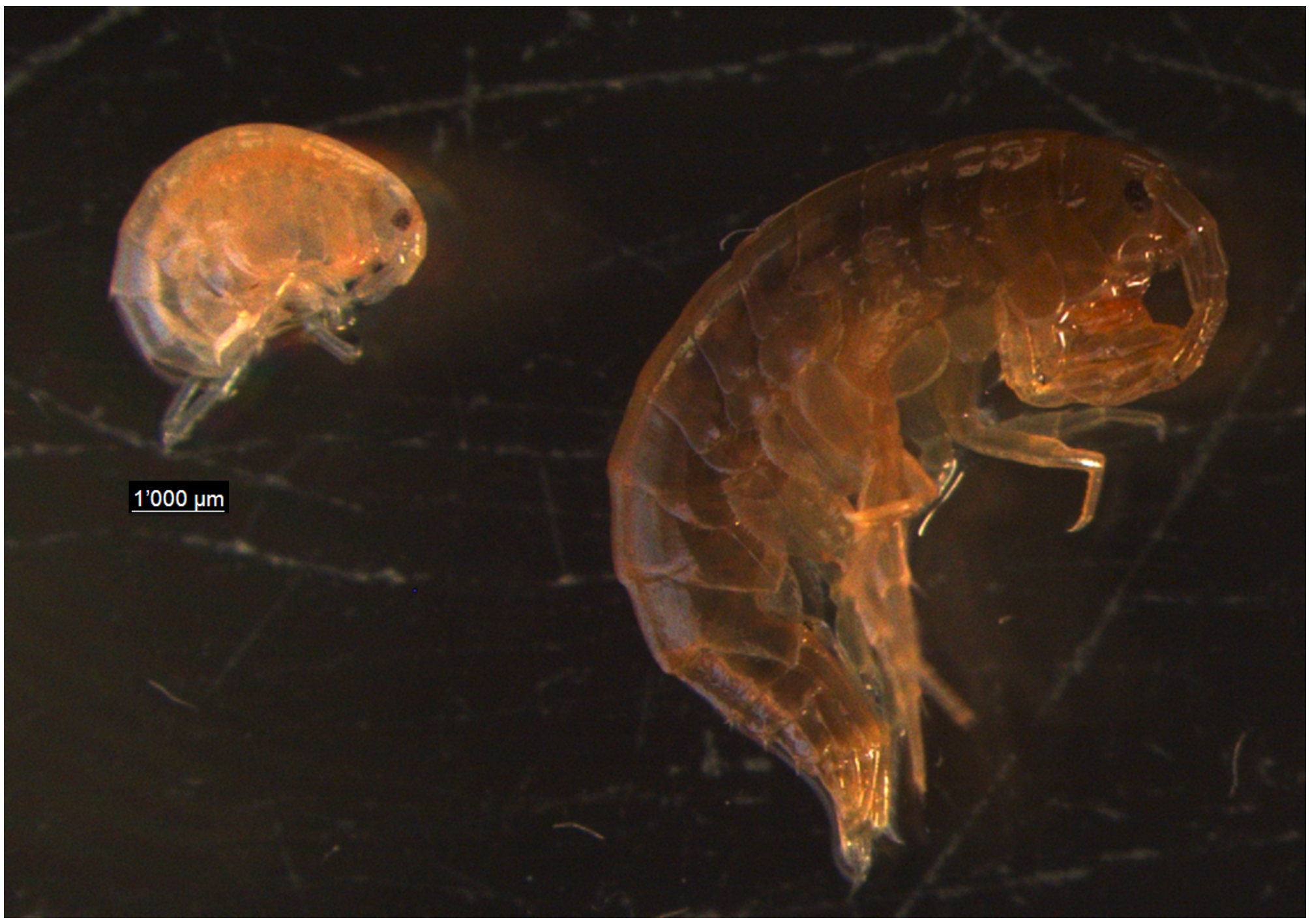

Figure SI-A1: Two amphipod species, *H. azteca* (left) and *G. pulex* (right), were used for the experiments. Both specimens shown are mature individuals. *H. azteca* (5 months old) was cultured at Eawag Dübendorf, while *G. pulex* (right) was collected from the field in March 2025.

Table SI-A2: Experimentally determined lipid and protein content for *G. pulex* and *H. azteca* across batches. Reported means are calculated as the weighted mean across quantifications, accounting for the number of animals per replicate.
Total protein content was quantified using a mass-balance approach: rows reporting only a single total protein value were quantified directly from the combined pre-ultracentrifugation fractions (SN1 + SN2), whereas rows reporting both total protein and membrane protein content were determined by summing the separated post-ultracentrifugation supernatant (SN) and membrane protein (MP) fractions (SN1 + SN2 + MP).

**Date**	**Lipid content [% w.w.]**	Replicates (Animals/rep.)	**Total protein content [% w.w.]**	**Membrane protein content [% w.w.]**	Replicates (Animals/rep.)
G. pulex	1.7 ± 1.0		4.4 ± 0.8	0.8 ± 0.1	
December 23	0.9 ± 0.0	$n = 3$ (4)	3.7 ± 0.4		$n = 3$ (4)
			4.8 ± 1.2	0.6 ± 0.2	$n = 3$ (4)
April 24	3.7 ± 1.0	$n = 3$ (4)	5.7 ± 1.1		$n = 2$ (4)
			5.0 ± 0.1	0.8 ± 0.1	$n = 2$ $(1 \times 80, 1 \times 32)$
June 24	1.2 ± 0.3	$n = 3$ (4)	–	–	–
July 24	1.0 ± 0.2	$n = 3$ (4)	–	–	–
October 24	0.4 ± 0.1	$n = 3$ (4)	–	–	–
November 24	1.6 ± 0.1	$n = 3$ $(1 \times 3, 2 \times 4)$	5.7 ± 0.1	0.8 ± 0.1	$n = 3$ (4)
			3.7 ± 0.5	0.9 ± 0.1	$n = 2$ $(1 \times 110, 1 \times 30)$
January 25	1.7	$n = 1$ (4)	–	–	–
February 25	3.8 ± 0.8	$n = 4$ $(3 \times 4, 1 \times 5)$	–	–	–
March 25	3.1 ± 0.5	$n = 3$ (4)	–	–	–
August 25	1.1 ± 0.3	$n = 16$ $(1 \times 3, 15 \times 4)$	–	–	–
September 25	1.6 ± 0.5	$n = 6$ $(5 \times 4, 1 \times 9)$	–	–	–
October 25	1.8 ± 0.6	$n = 5$ $(3 \times 4, 2 \times 12)$	–	–	–
November 25	2.1 ± 0.3	$n = 7$ $(5 \times 4, 2 \times 10)$	–	–	–
H. azteca	3.3 ± 0.2		6.2 ± 0.9	0.9 ± 0.2	
November 24	3.3 ± 0.2	$n = 3$ (10)	8.0 ± 0.8	1.1 ± 0.2	$n = 3$ (10)
January 25	–	–	9.6 ± 0.2		$n = 3$ $(2 \times 10, 1 \times 11)$
			7.1 ± 0.4	0.6 ± 0.0	$n = 3$ (10)
				0.6	$n = 1$ (92)
			6.7	1.3	$n = 1$ (161)
November 25	–	–		0.8 ± 0.0	$n = 2$ (331, 584)
February 26	–	–	5.7	0.9	$n = 1$ (643)

Wet weight–dry weight ratios were determined as the ratio of wet weight to dry weight, following the protocol described elsewhere.[6] Dry weight was determined after organisms were euthanized in ethanol, placed in pre-weighed glass vials, and dried overnight at 60 °C. Ratios are similar to previous findings.[6]

Table SI-A3: Experimentally determined wet and dry weight conversion factors for *G. pulex* and *H. azteca*.

**Species**	**Wet/dry weight ratio**	**Replicates**	**Animals per replicate**
G. pulex	4.8 ± 0.3	$n = 3$	4
H. azteca	4.1 ± 0.1	$n = 5$	10

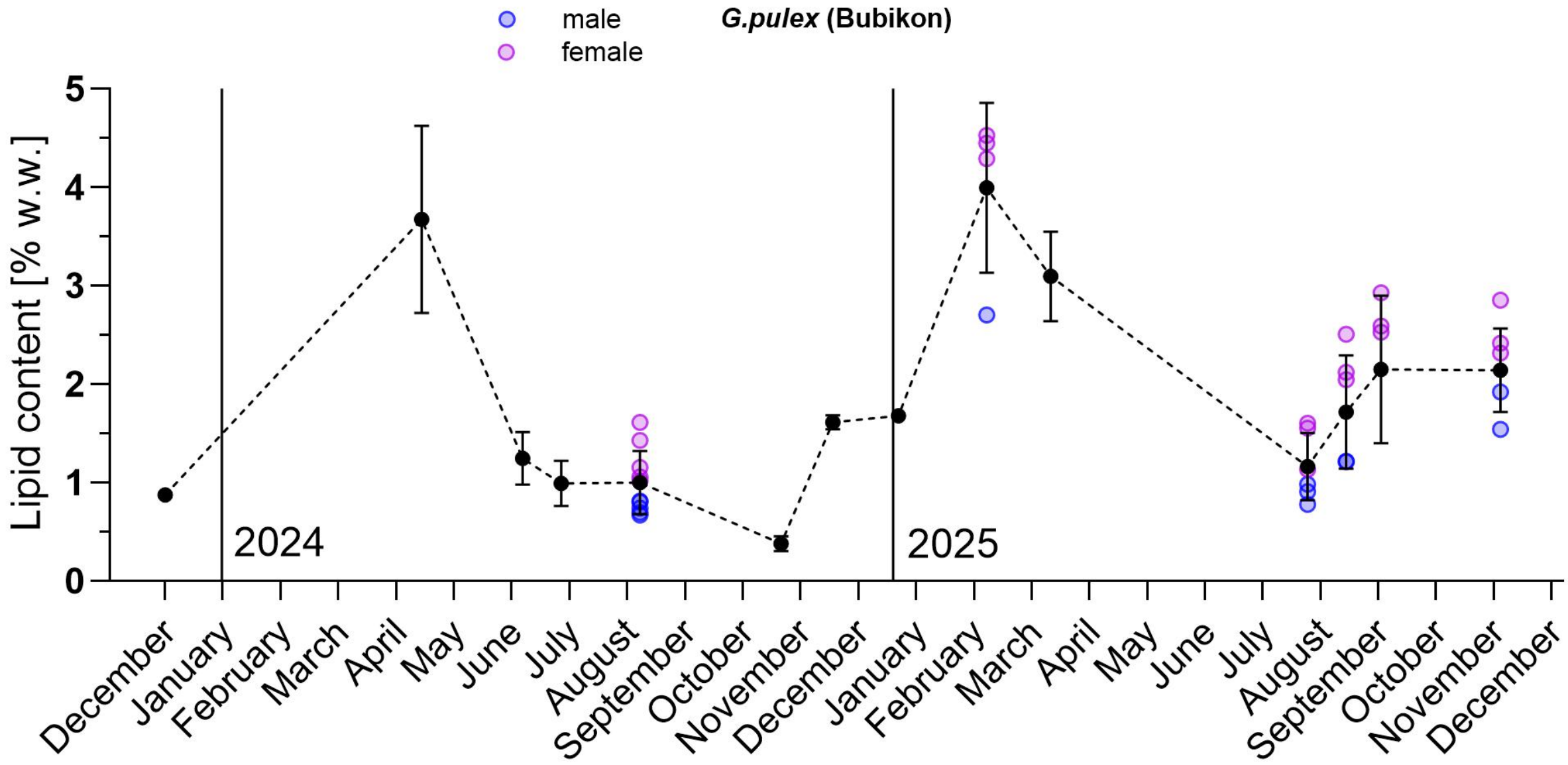

Figure SI-A2: Seasonal fluctuations of mean lipid content for *G. pulex* over two years. At specific times, samples were distinguished between males (blue) and females (pink). All genders are pooled (black), the mean ± SD does not account for the number of animals per replicate.

Collections during the year reflect seasonal fluctuations (Figure SI-A2) in the population of *G. pulex* and higher lipid content in female organisms as similarly reported by Sroda and Cossu-Leguille[7] and Dalhoff et al.[8], who concluded that food availability and temperature influence energy reserves, with maximum levels during fall and winter. Our findings show a maximum in early spring and a minimum during summer, which could reflect greater locomotion with higher metabolic rates. However, because we did not sample at continuous monthly intervals, we cannot draw a definitive conclusion regarding the full seasonal cycle. Geographical differences or reproductive cycles could account for the differences observed, though the overall lipid range we observed is similar to reported values.[8]

Comparing *G. pulex* and *H. azteca* (Table SI-A2), Raths et al.[6] also observed, higher lipid content and higher protein content for *H. azteca* relative to *G. pulex*.

SI-A-S3 Experiments and Endpoints

Table SI-A4: Overview of the experiments performed. The experiment numbers correspond to those listed in SI-B.

Experiment	Compounds	Nominal concentration	Duration [days]		Extraction / Analysis	Date
		[μg L^{-1}]	Total	Exposure phase		
G. pulex						
G_1	IMI, FPF, ACE	10, 10, 5	14	3	Toxicokinetic	December 2023
G_2	IMI, FPF, ACE, THI	50, 25, 25, 25	6	3, 3, 3, 2	*In-vivo* assay	March 2024
G_3	SPI	50	18	3	Toxicokinetic	April 2024
G_4	IMI	50	6	3	*In-vivo* assay	July 2024
G_5	IMI	50	6	3	Dissection	November 2024
G_6	SPI	25	6	3	*In-vivo* assay	December 2024
G_7	IMI, FPF, ACE, THI<br>IMI-ole	0.2 – 10	–	–	*In-vitro* assay	July 2025<br>February 2026
H. azteca						
H_1	FPF, THI	10	18	3	Toxicokinetic & *In-vivo* assay	October 2024
H_2	IMI, THI	25	6	3	*In-vivo* assay	November 2024
H_3	ACE, FPF	5, 10	6	3	*In-vivo* assay	November 2024
H_4	ACE, IMI	5, 10	18	3	Toxicokinetic & *In-vivo* assay	January 2025
H_5	THI,<br>IMI, FPF, ACE<br>THI, IMI-ole	0.2 – 10	–	–	*In-vitro* assay	January 2025<br>February 2026

SI-A-S3.1 Toxicity and Mortality

Toxicokinetic exposure concentrations (max. 3-day exposure phase) were kept below literature values for acute toxicity (Table SI-A5). The only exception was *H. azteca* exposed to ACE at >5 μg L^{-1} (mortality 11.1%, Table SI-A6). However, literature toxicity values were reported for juvenile *H. azteca*, while adult organisms exhibited lower sensitivity in this study.
For Spinosad, no literature data was available.

Table SI-A5: Literature-derived LC_{50} values for selected insecticides.

Species	THI [μg L^{-1}]	IMI	ACE	FPF	Endpoint tested	Source
G. pulex	350	270	50	–	LC_{50}, 96h	Beketov and Liess[9]
G. pulex	–	731	–	112	LC_{50}, 72h	Huang et al.[10]
G. pulex	580	–	–	–	LC_{50}, 96h	Beketov and Liess[11]
H. azteca	55	360	4.8	–	LC_{50}, 96h	Raby et al.[12]
H. azteca	68	230	4.7	26	LC_{50}, 7 days (juveniles)	Bartlett et al.[13]

Table SI-A6: Experimental conditions across experiments. The experiment numbers correspond to those listed in SI-B.

Experiment	Compound	Mortality [%]	Exposure concentration [μg L^{-1}]	Experiment duration [d]	pH	Temperature [°C]
G. pulex						
G_1	ACE	15.9	5.1	14	7.4 ± 0.1	15.2 ± 0.2
G_2	ACE	20.8	22.1	6	7.6 ± 0.3	15.2 ± 0.2
G_1	FPF	14.0	11.6	14	7.4 ± 0.2	15.2 ± 0.2
G_2	FPF	9.1	27.2	6	7.6 ± 0.3	15.2 ± 0.2
G_1	IMI	15.9	10.7	14	7.3 ± 0.1	15.2 ± 0.2
G_2	IMI	20.5	58.4	6	7.6 ± 0.3	15.2 ± 0.2
G_5	IMI	15.0	33.3	6	7.6 ± 0.1	14.8 ± 0.6
G_4	IMI	15.4	46.3	6	7.7 ± 0.2	*NA*
G_3	SPI	11.3	47.8	18	7.6 ± 0.3	15.3 ± 0.3
G_6	SPI	10.5	20.6	6	7.6 ± 0.3	15.0 ± 0.1
G_2	THI	4.6	23.3	5	7.5 ± 0.3	15.2 ± 0.2
H. azteca						
H_3	ACE	9.4	3.0	6	7.9 ± 0.1	20.9 ± 0.3
H_4	ACE	11.1	5.6	18	7.7 ± 0.3	20.4 ± 0.5
H_1	FPF	4.6	9.1	18	7.6 ± 0.1	22.0 ± 0.4
H_3	FPF	8.7	8.1	6	7.8 ± 0.1	20.9 ± 0.3
H_2	IMI	10.0	20.1	6	8.0 ± 0.1	20.9 ± 0.3
H_4	IMI	15.5	15.2	18	7.7 ± 0.3	20.4 ± 0.5
H_1	THI	13.9	10.5	18	7.6 ± 0.1	22.0 ± 0.4
H_2	THI	10.0	23.8	6	8.0 ± 0.1	20.9 ± 0.3

SI-A-S3.2 Dissection method

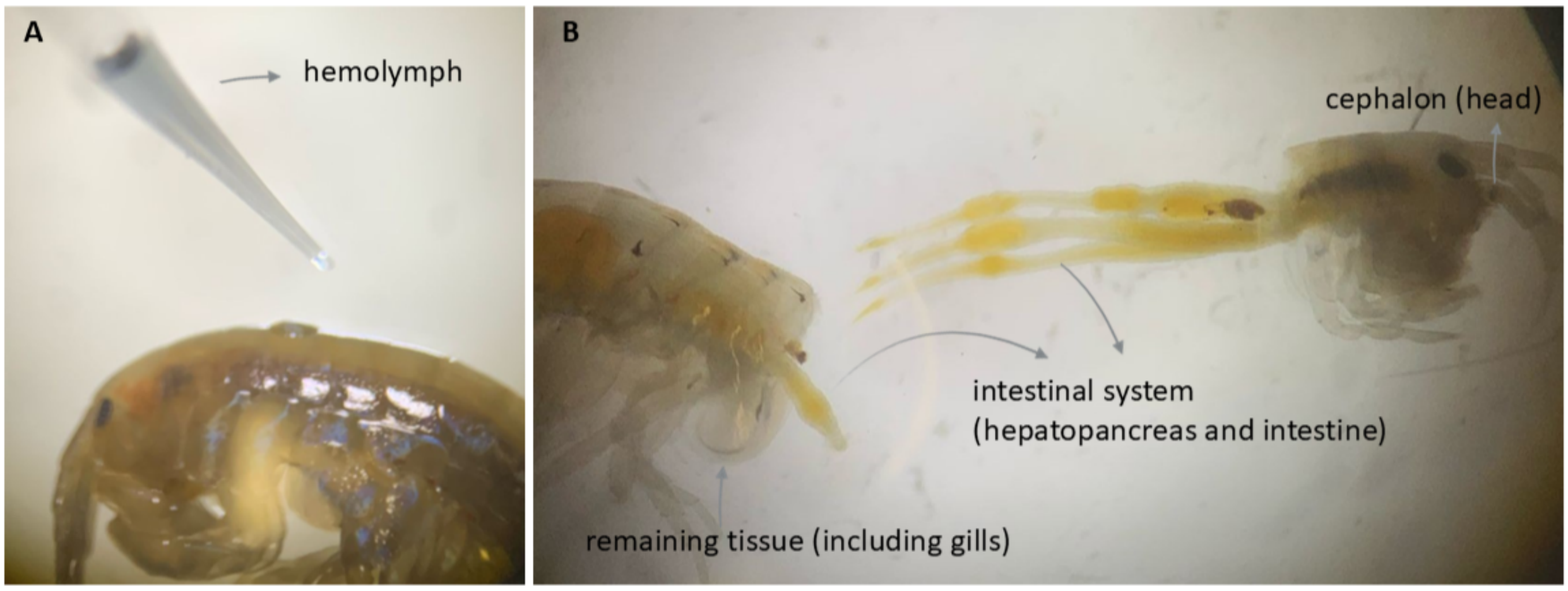

Figure SI-A3: Microscopic images showing the hemolymph extraction (A) and the dissection (B) of *G. pulex* for the sub-organismal distribution analysis. Hemolymph was extracted by inserting a capillary needle into the dorsal region. The dissection was performed by separating the cephalon from the carcass and pulling the gut through the cephalic end.

SI-A-S3.3 *In vitro* assay

The *in vitro* assay was independently performed with two species (Table SI-A7). Next to *G. pulex* from the Swiss population (Location: 47.274900, 8.789200), a population from the Netherlands was used. Organisms were collected from an uncontaminated location, the Heelsumse Beek (a brook with the coordinates 51.973400 °N, 5.748697 °E), on 26.11.2024 by Anna Huang. For the NL population, we quantified the total protein content at 4.5% wet weight and the membrane protein content at 0.7% wet weight.
For *H. azteca*, the FPF assay was performed once with 0.7 ml and repeated with 1 ml of MP extract to validate that the protein quantity didn't limit the assay in regard to the limit of quantification.
While determining the MP recovery, we observed 44% and 21% recoveries after the first and second ultracentrifuge steps, respectively. The protein content in the supernatant was <LOQ. Thus, no mass balance quantification was possible.
The MP recovery rate published by Raths et al.[5] was 24% throughout the *in vitro* assay.

Table SI-A7: Details of the independent *in vitro* assay experiments performed with species-specific membrane protein (MP) preparation. Each compound concentration was tested in duplicate ($n = 2$) split over multiple runs of 8 vials each.

Species	Location	Compounds	Protein content [mg]	Quantity of MP extract [ml]	Pooled animals: weight [mg] / organisms	Relative Recovery [%]
G. pulex	Field, CH	THI / IMI<br>ACE / FPF	0.16	0.5	2.95×10^3 mg	21
		IMI-ole	0.32	1.0	$n = 172$	
G. pulex	Field, NL	THI / FPF	0.16	0.5	6.93×10^2 mg	–
		IMI-ole	0.25	0.8	–	
H. azteca	Culture	THI	0.45	0.4	7.54×10^2 mg<br>$n = 253$	25
H. azteca	Culture	THI / IMI<br>ACE / FPF	0.43	0.7	1.84×10^3 mg	50
		FPF / IMI-ole	0.61	1.0	$n = 643$	

SI-A-S4 Chemical measurement

Table SI-A8: Online-SPE setup on the QExactive Plus mass spectrometer.
The online SPE column was prepared by adding 8 mg to 9 mg of Oasis HLB (15 µm particle size, Waters) and about 8 mg to 9 mg of a mix of an anion exchanger Strata X-AW, cation exchanger Strata X-CW (both 30 µm, Phenomenex, UK) and Env+ (70 µm, Biotage, Sweden) in a ratio of 1:1:1.5 (X-AW : X-CW : Env+) to an empty stainless steel SPE cartridge (20 x 2.1 mm, BGB Analytik AG).

Time [min]	Acetonitrile [µLmin^{-1}]	Ammonium acetate solution (2 mM) [µLmin^{-1}]	SPE step
0	—	200	Elution of the sample from the cartridge (with elution pump) and washing of the loop.
0.1	4000	—	
1.1	4000	—	
1.2	—	4000	
6.7	—	4000	
6.8	—	400	
7.3	—	400	
7.4	400	—	Loading of the new sample into the loop and conditioning of the cartridge.
12.5	400	—	
12.6	—	400	
18.4	—	400	
18.5	—	1270	Enrichment of the new sample.
32.1	—	1270	
34.5	—	1270	
34.7	—	1270	
35	—	200	

Table SI-A9: Gradient of the liquid chromatography. Water and methanol were both acidified with 0.1% (vol.) formic acid.

Time [min]	H_2O [µLmin^{-1}]	MeOH [µLmin^{-1}]
0.0	0.260	0.040
5.0	0.260	0.040
20.0	0.015	0.285
29.0	0.015	0.285
29.5	0.260	0.040
35.0	0.260	0.040

Table SI-A10: Source and MS parameters used for HRMS/MS measurement with the QExactive Plus mass spectrometer.

Parameter	Value
Sheath gas (nitrogen) flow rate	40 L min^{-1}
Auxiliary gas (nitrogen) flow rate	15 L min^{-1}
Capillary temperature	320 ℃
S-lens RF level	50
Mass calibration	External (acid solution -11 amino acids (m/z between 116 and 997)
Spray voltage	4 kV (positive ionisation mode)
Resolution	MS1: 70k | MS2 (isolation window: 1 m/z): 30k
Scan range	150–2000 m/z
Polarity	Positive

SI-A-S5 Toxicokinetic model

SI-A-S5.1 Model parameters

Model scripts of the main model were previously provided on GitHub (`https://github.com/NikaGoldring/TK-receptor`).
The adapted model (including growth) is provided on GitHub (`https://github.com/HollenderGroup/Bioaccumulation_TK-receptor_growth`).

Note: Internal concentrations derived from long-duration toxicokinetic experiments (>10 days) with multiple time points were used to calibrate the model. Afterward, concentrations from shorter toxicokinetic experiments (<6 days) with two time points were used to validate. We acknowledge that calibrating with one concentration and validating a toxicokinetic model using datasets restricted to two time points, limits quantitative precision and recommend that future work utilize higher-resolution time-series validations and calibration sets across concentrations. In this study, we prioritized capturing receptor-binding patterns across different compounds and species.

Table SI-A11: Parameter symbols and explanations for the TK-receptor model.

Symbol	Explanation	Unit	Model: Fitted / fixed
C_w	Exposure concentration	μM	forcing, exposure scenario
FMS	Factor to normalize for membrane protein content (density assumption of 1 kg L^{-1})	kg$_{\text{MP}}$ kg$^{-1}_{\text{Structure}}$	fixed:<br>*G. pulex*: 0.008<br>*H. azteca*: 0.009
g_t	Growth rate	d^{-1}	fixed:<br>*G. pulex*: –<br>*H. azteca*: 0.059
State variables			
C_S	Concentration of ligand in structure compartment	μmol kg$^{-1}_{\text{Structure}}$	state variable
C_{MP}	Concentration of ligand in membrane protein compartment	μmol kg$^{-1}_{\text{MP}}$	state variable
C_{Total}	Total tissue concentration	μmol kg^{-1}	algebraic sum
Parameter			
k_u	Uptake rate constant	L kg$^{-1}_{\text{S}}$ d^{-1}	
k_e	Elimination rate constant	d^{-1}	
k_{on}	Association rate constant for ligand-receptor complex	kg$_{\text{S}}$ kg$^{-1}_{\text{MP}}$ d^{-1}	Fixed to 0 (1-compartment) or 200 (2-compartment)
k_{off}	Dissociation rate constant of ligand-receptor complex	d^{-1}	fixed to 0
B_{max}	Maximum binding capacity	μmol kg$^{-1}_{\text{MP}}$	
Model evaluation			
AIC	Akaike Information Criterion	–	–
R^2	Coefficient of determination	–	–
$\%Bias$	Relative Bias	–	–
$RMSE$	Root Mean Square Error	–	–
CCC	Concordance Correlation Coefficient	–	–

SI-A-S5.2 Growth considerations

Our data over an extended temporal scale (compared to Raths et al.[5]) indicate a slow but sustained decline in concentration during elimination, particularly visible after day 10. When we compared the absolute body burden (total mass of the chemical) over time, rather than the internal concentration normalized to the body weight, the total amount of chemical remained stable (Figure SI-A4).
While we observed a consistent growth rate across experiments for *H. azteca*, no growth rate could be determined for *G. pulex* (Figure SI-A5). Variations between laboratory and field organisms likely cause this discrepancy. We attribute this difference to a homogeneous developmental stage among laboratory-cultured *H. azteca* individuals, in contrast to the age variability among field-collected *G. pulex*.
For *H. azteca*, 10 individuals were analyzed per time point, whereas only four were used for *G. pulex*, resulting in higher variability in the dataset. Additionally, notable sex-related differences in body weight, which were not accounted for during *G. pulex* sampling, may have contributed to the variability. We had no growth data for a control group, yet published growth data (size, mm)[10] for *G. pulex* over 28 days, shows to be independent of tested exposure concentration (i.e., 0–30 μg L^{-1}).
For model implementations, we considered using von Bertalanffy growth dynamics used in the Dynamic Energy Budget (DEB) theory and are aware that growth not only dilutes internal concentration but also affects the elimination rate constant.[14,15] Yet, as our goal was to construct a generalized model without the complexity of a fully size-dependent, mechanistic model, we simplified by adding a first-order growth rate constant, $g(t)$ (d^{-1}), and determining the growth rate across all experiments to minimize the input parameters needed for the model set-up. The growth rate (0.059 (0.045–0.073)) was determined using an exponential growth (log) model, $Y = \log Y_0 + k \cdot X$.

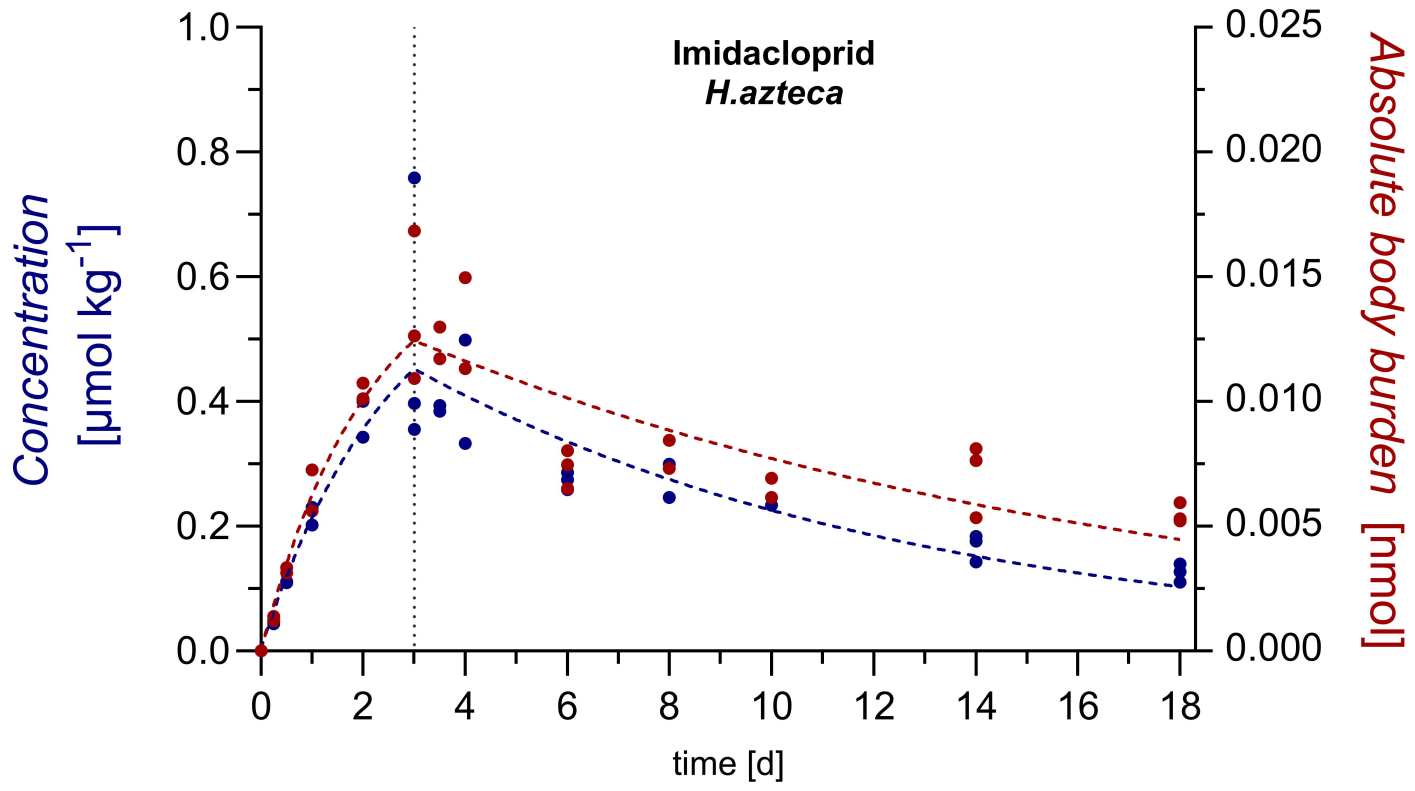

Figure SI-A4: Comparison of measured imidacloprid concentration (μmol kg^{-1}; left y-axis, blue) and absolute body burden (nmol; right y-axis, red) in replicates from *H. azteca* over 18 days with a simple association and dissociation kinetic fit. The internal concentration (C) represents the absolute body burden (M) normalized by the wet weight (w.w.) of the replicate ($C = M/$w.w.), highlighting the influence of the weight.

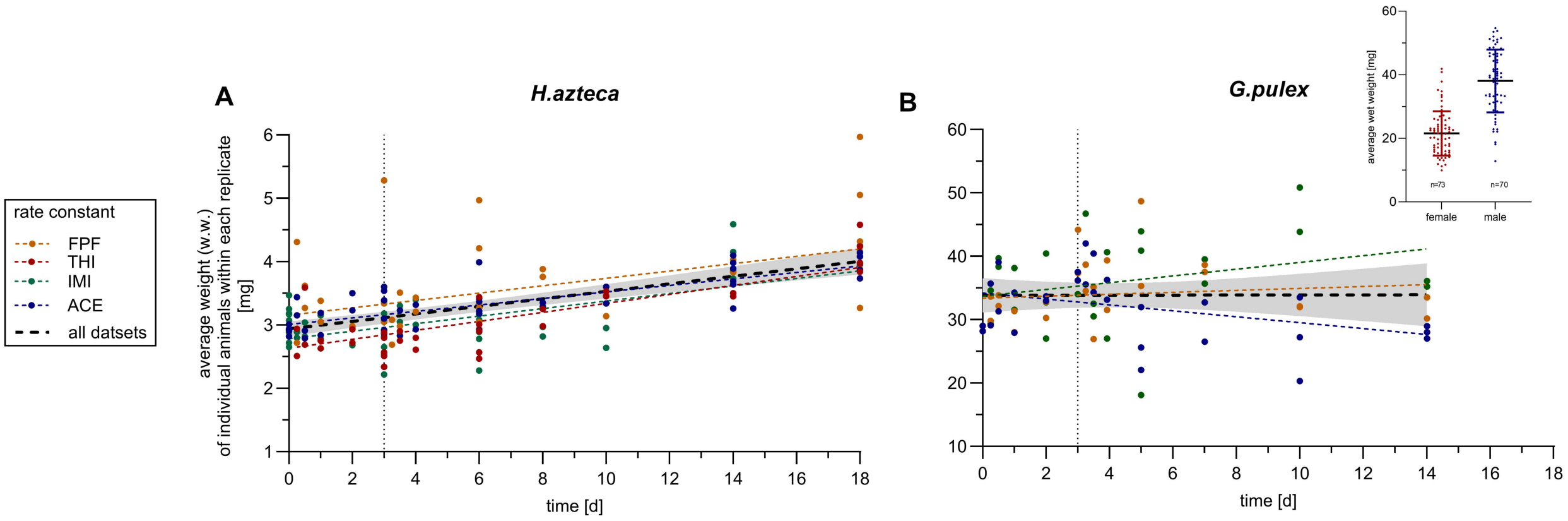

Figure SI-A5: Mean wet weight (w.w.) of an individual organism within each replicate for A) *H. azteca* and B) *G. pulex* at each sampling time point of each long-duration experiment conducted (THI = thiacloprid in red, FPF = flupyradifurone in orange, IMI = imidacloprid in green, and ACE = acetamiprid in blue). The black dotted line represents the combined fit for growth across all experiments, including the 95 % confidence interval (in shaded gray). The growth rate (rate constant) was determined by fitting exponential growth with log(population) $Y = \log Y_0 + k \cdot X$. The vertical dashed line indicates the transition between the uptake and elimination (the period with food application) phases.

SI-A-S5.3 Two-compartment model

Model complexity considerations:
Two-compartmental toxicokinetic model, considering the effect of growth and dissociation, tested for *H.azteca* exposed to THI (Figure SI-A12):
We evaluated the relative contributions of growth dilution ($g_t > 0, k_{\text{off}} = 0$) and receptor dissociation ($k_{\text{off}} > 0, g_t = 0$) and their combined effect ($g_t, k_{\text{off}} > 0$) to the observed slow elimination. Growth dilution accounted for nearly $100\,\%$ of the contaminant loss during the depuration phase, with the remaining minimal variance attributable to growth rate fluctuations (0.045–0.073 d^{-1}). Consequently, our data lack the sensitivity required to distinguish specific substrate-receptor dissociation kinetics.
Integrating additional model complexity would lead to overfitting rather than improving biological insights, as the data is not sufficiently sensitive to disentangle dissociation kinetics from growth effects. Thus, for simplicity, the first-order rate ($k_{\text{off}} = 0$ d^{-1}) was neglected and set to 0, assuming irreversible receptor binding for all neonicotinoids (Figures SI-A6–SI-A11). The simplified model was selected because it provided a statistically robust explanation for the observed elimination (e.g., based on an AIC of -41). For k_{on}, the default parameter from Raths et al.[5] was chosen.
The equation applied was:

$$
\begin{aligned}
\frac{dC_{\text{MP}}(t)}{dt} &= k_{\text{on}} \cdot C_S(t) \cdot (B_{\text{max}} - C_{\text{MP}}(t)) - k_{\text{off}} \cdot C_{\text{MP}}(t) - g(t) \cdot C_{\text{MP}}(t) \\
\frac{dC_S(t)}{dt} &= k_u \cdot C_w(t) - k_e \cdot C_S(t) - \frac{dC_{\text{MP}}(t)}{dt} \cdot FMS - g(t) \cdot C_S(t) \\
C_{\text{tot}}(t) &= C_S + C_{\text{MP}} \cdot FMS
\end{aligned}
\tag{1}
$$

Table SI-A12: Comparison of the two-compartment model evaluating growth, receptor dissociation, and both parameters for *H. azteca* exposed to Thiacloprid, including model fits, parameter estimates, with the equation 1.

**Parameter**	**Growth** ($g_t > 0, k_{\text{off}} = 0$)	**Dissociation** ($k_{\text{off}} > 0, g_t = 0$)	**Growth & dissociation** ($g_t, k_{\text{off}} > 0$)
AIC	-41.0461	-38.5636	-39.0514
R^2	0.93	0.93	0.93
Time	1 h, 20 min	7 h, 51 min	7 h, 33 min
k_e	1.726	3.508	1.788
k_u	9.713	9.722	9.718
k_{on}	200 – fixed	200 – fixed	200 – fixed
k_{off}	0 – fixed	0.8594	0.01262
B_{max}	63.42	74.89	64.09
g_t	0.059 – fixed	0 – fixed	0.059 – fixed

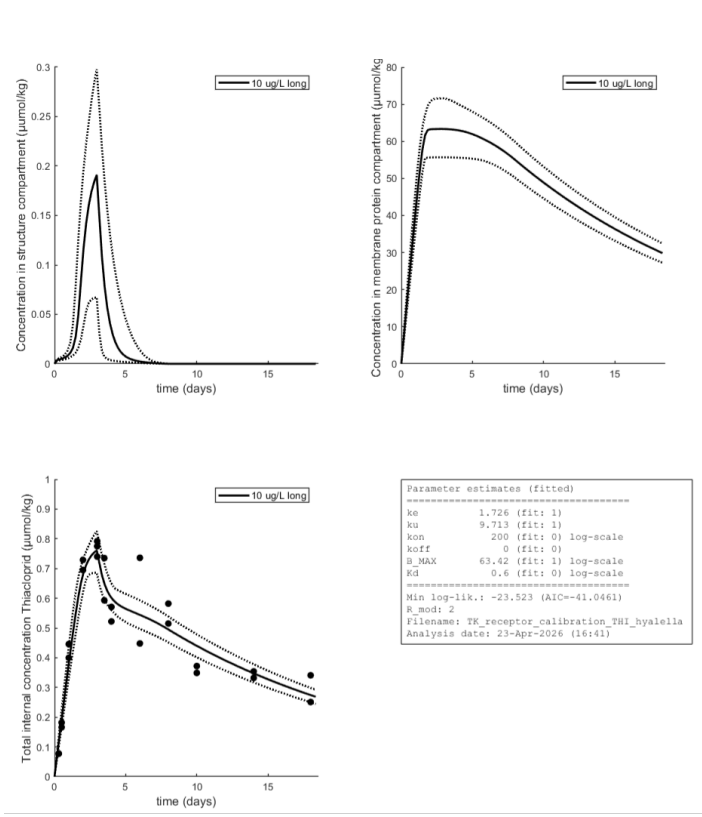

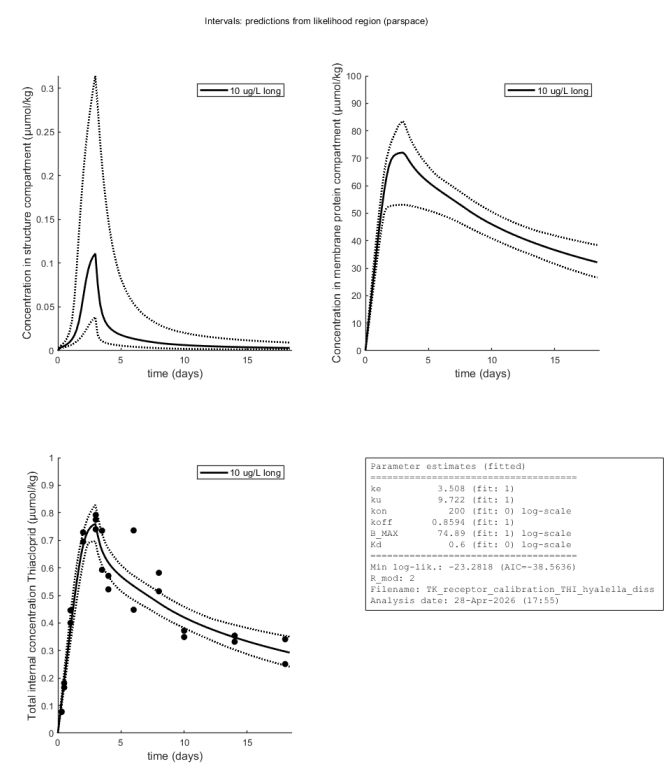

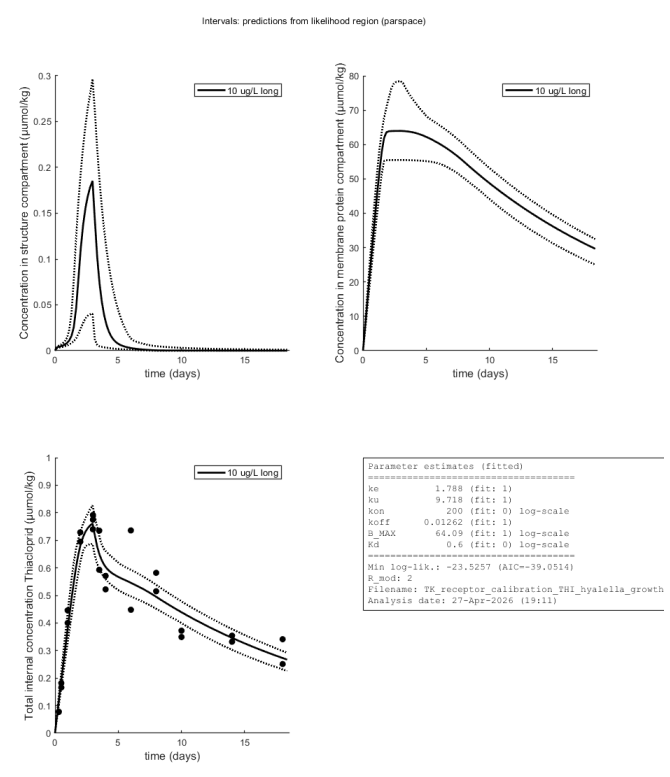

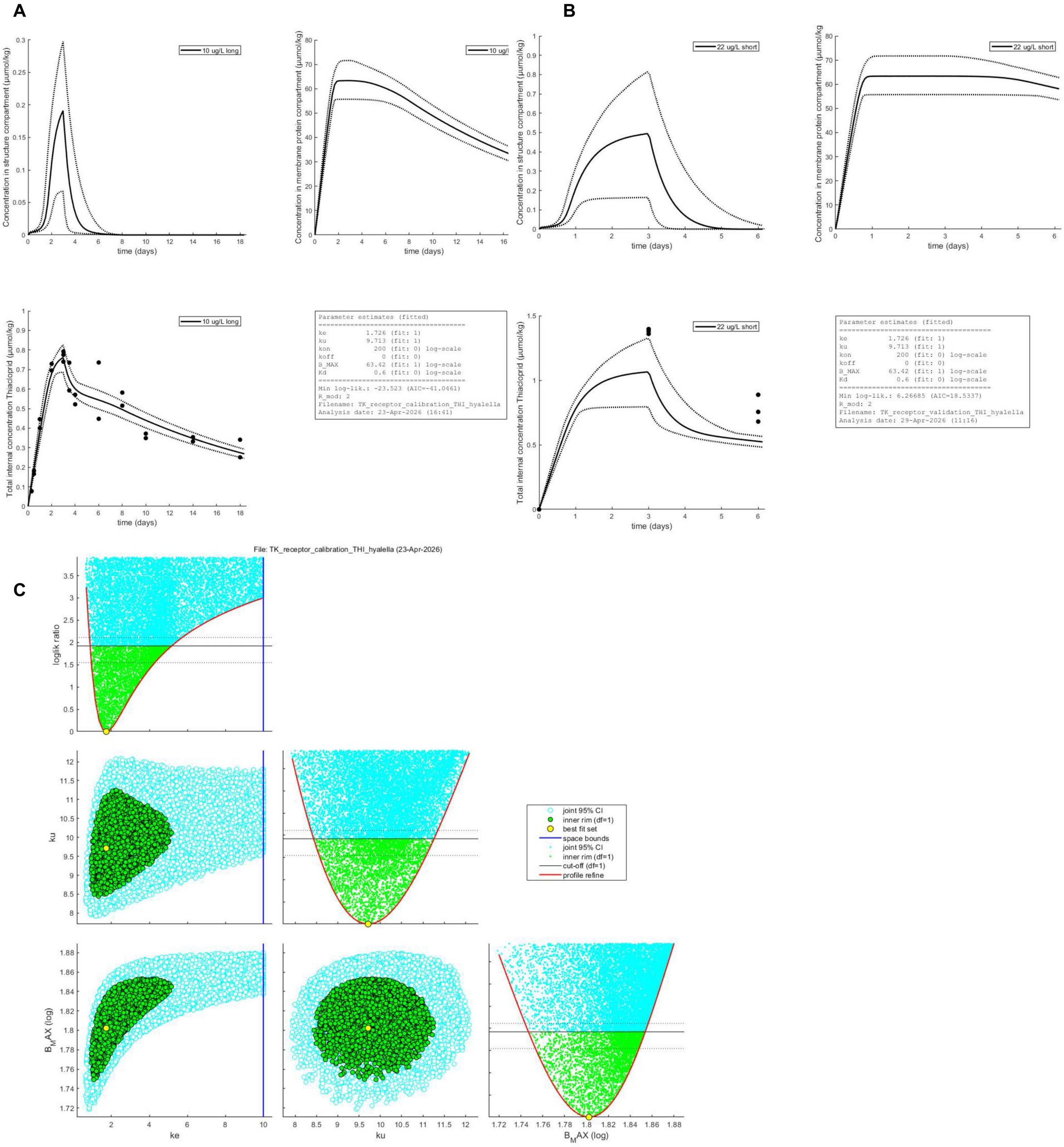

Figure SI-A6: Plots of (A) the model calibration, (B) the model validation, and (C) the parameter space of the calibration set for *H. azteca* exposed to Thiacloprid.

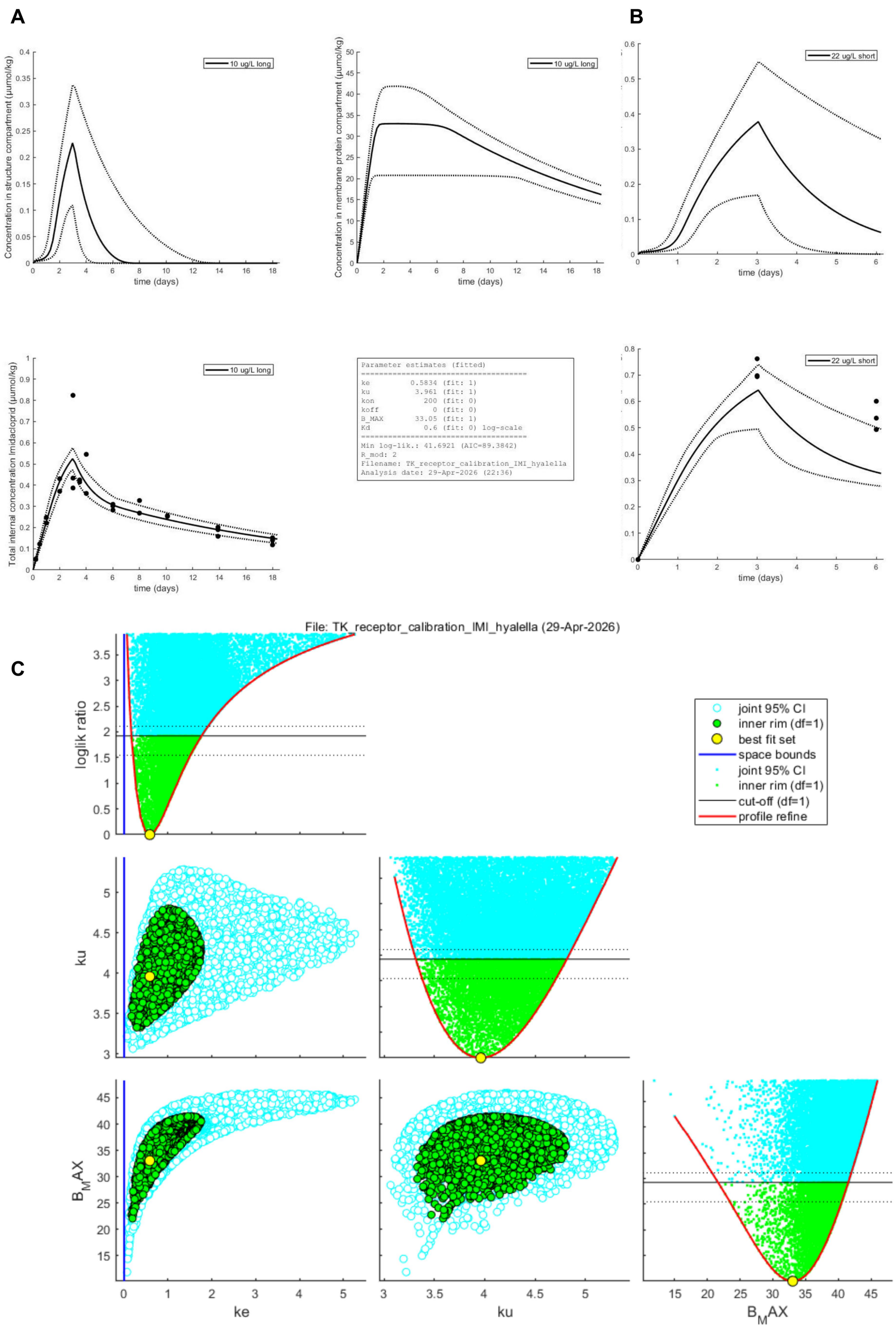

Figure SI-A7: Plots of (A) the model calibration, (B) the model validation, and (C) the parameter space of the calibration set for *H. azteca* exposed to Imidacloprid.

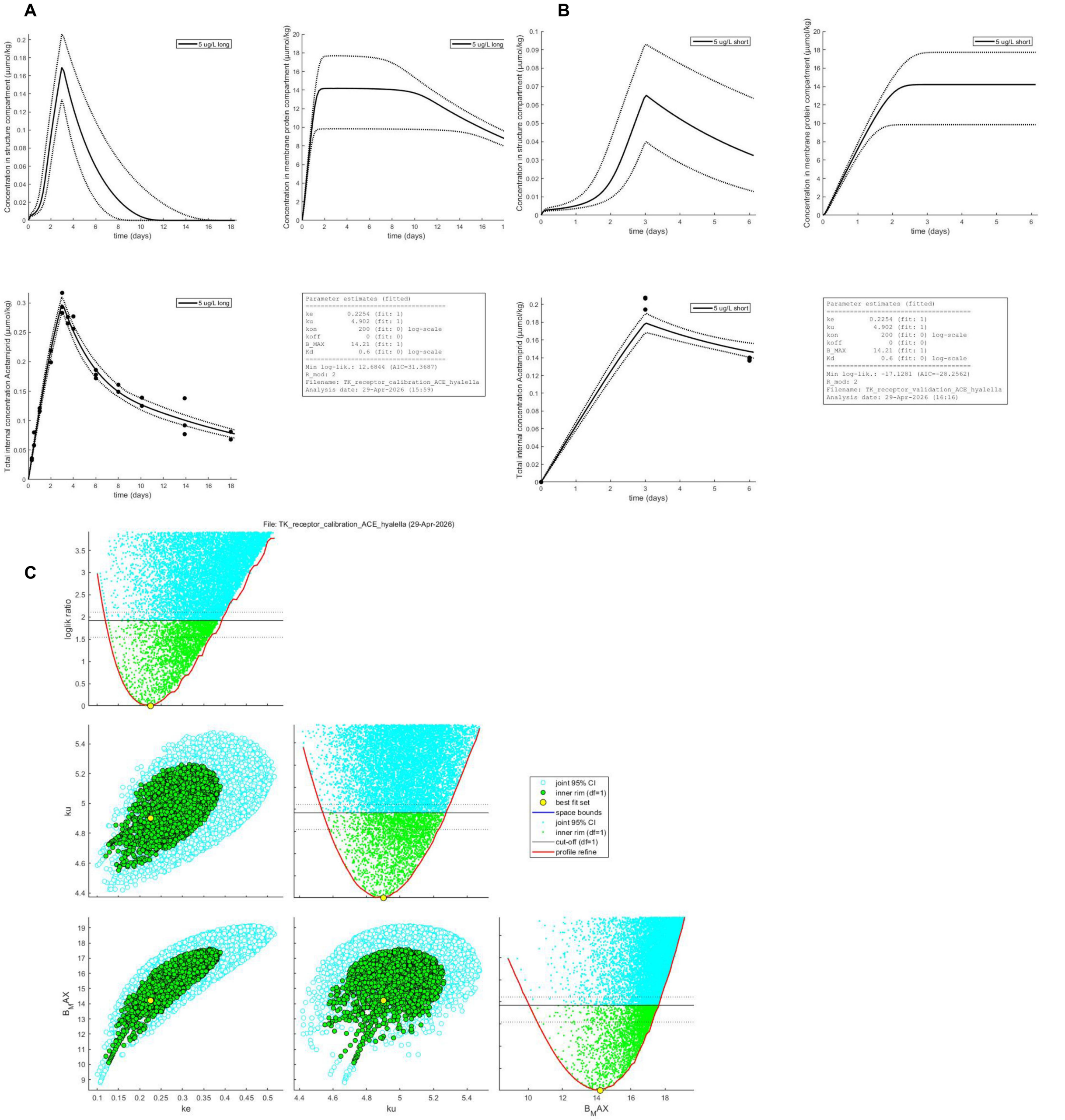

Figure SI-A8: Plots of (A) the model calibration, (B) the model validation, and (C) the parameter space of the calibration set for *H. azteca* exposed to Acetamiprid.

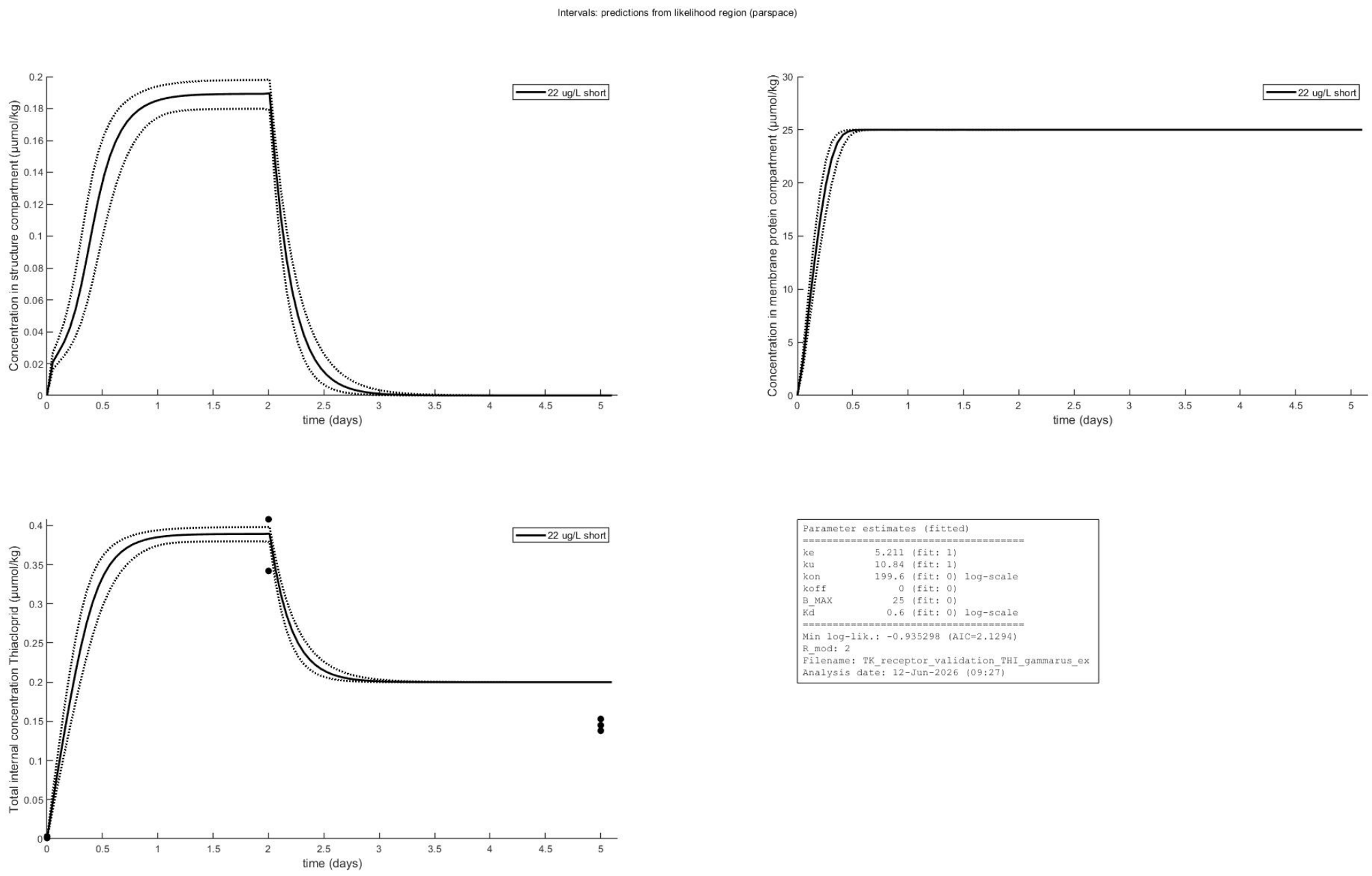

Figure SI-A9: Plot of the model validation for *G. pulex* exposed to Thiacloprid. The calibration and the parameter space of the calibration is published in Raths et al.[5].

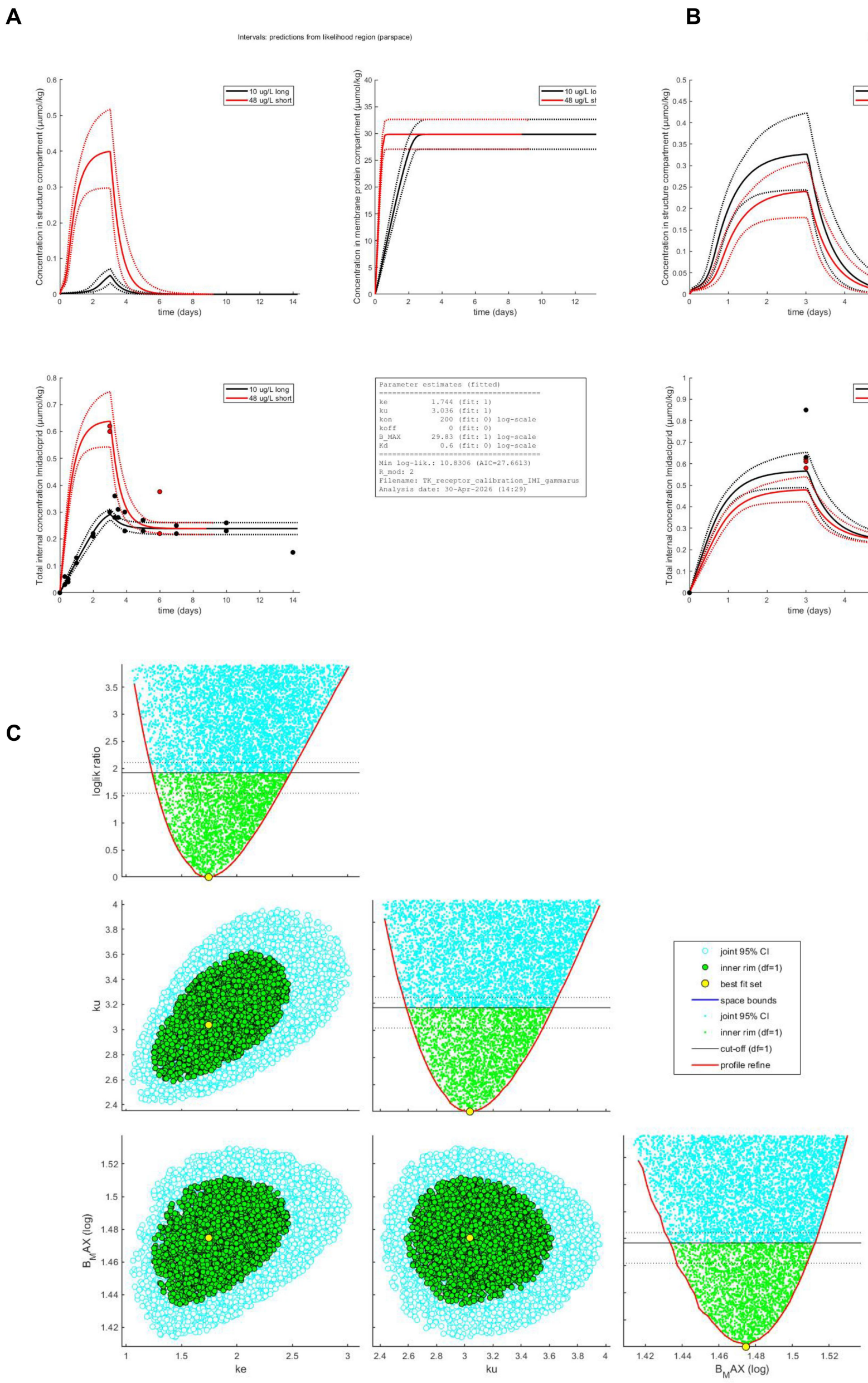

Figure SI-A10: Plots of (A) the model calibration, (B) the model validation, and (C) the parameter space of the calibration set for *G. pulex* exposed to Imidacloprid.

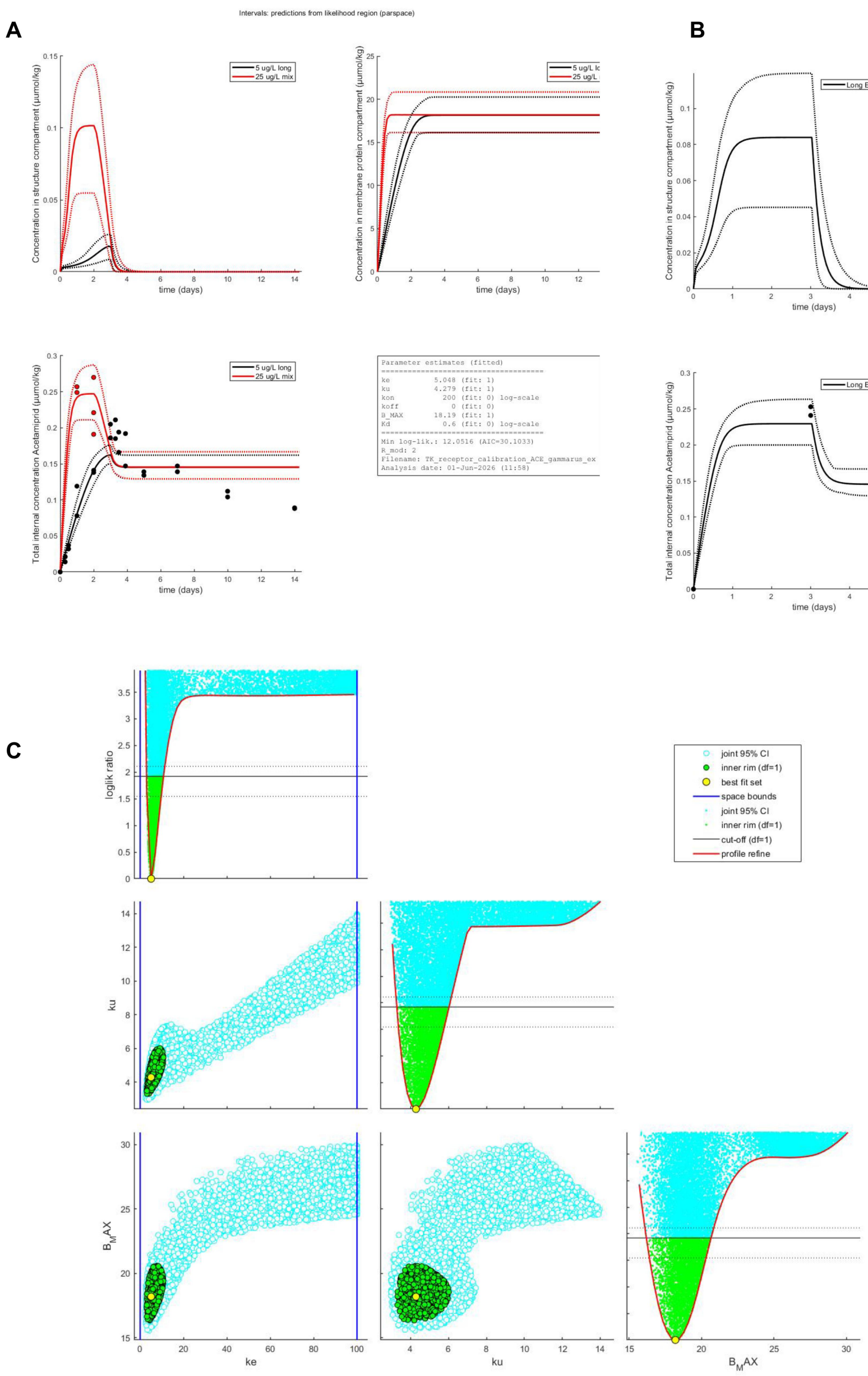

Figure SI-A11: Plots of (A) the model calibration, (B) the model validation, and (C) the parameter space of the calibration set for *G. pulex* exposed to Acetamiprid.

SI-A-S5.4 One compartment model

Model complexity considerations:
One-compartment vs. two-compartment toxicokinetic models tested for FPF in *G. pulex*: We chose the simplest model fit (i.e., ΔAIC $= 4.05$) for our purposes, i.e., the first-order, one-compartment model (eq. 2), for eliminating compounds (Figures SI-A12–SI-A14).

$$\frac{dC_S(t)}{dt} = k_u \cdot C_w(t) - k_e \cdot C_S(t) \tag{2}$$

Table SI-A13: Comparison of a one-compartment (eq. 2) with a two-compartment (eq. 1) model for *G. pulex* exposed to Flupyradifurone (FPF).

Parameter	One-compartment	Two-compartment
AIC	-49.92	-45.87
R^2	0.89	0.89
Time	12 min	1 h, 49 min
k_e	0.6002	0.5802
k_u	1.707	1.676
k_{on}	0 – fixed	200 – fixed
k_{off}	0 – fixed	50.47
B_{max}	0 – fixed	1.02×10^{-5}

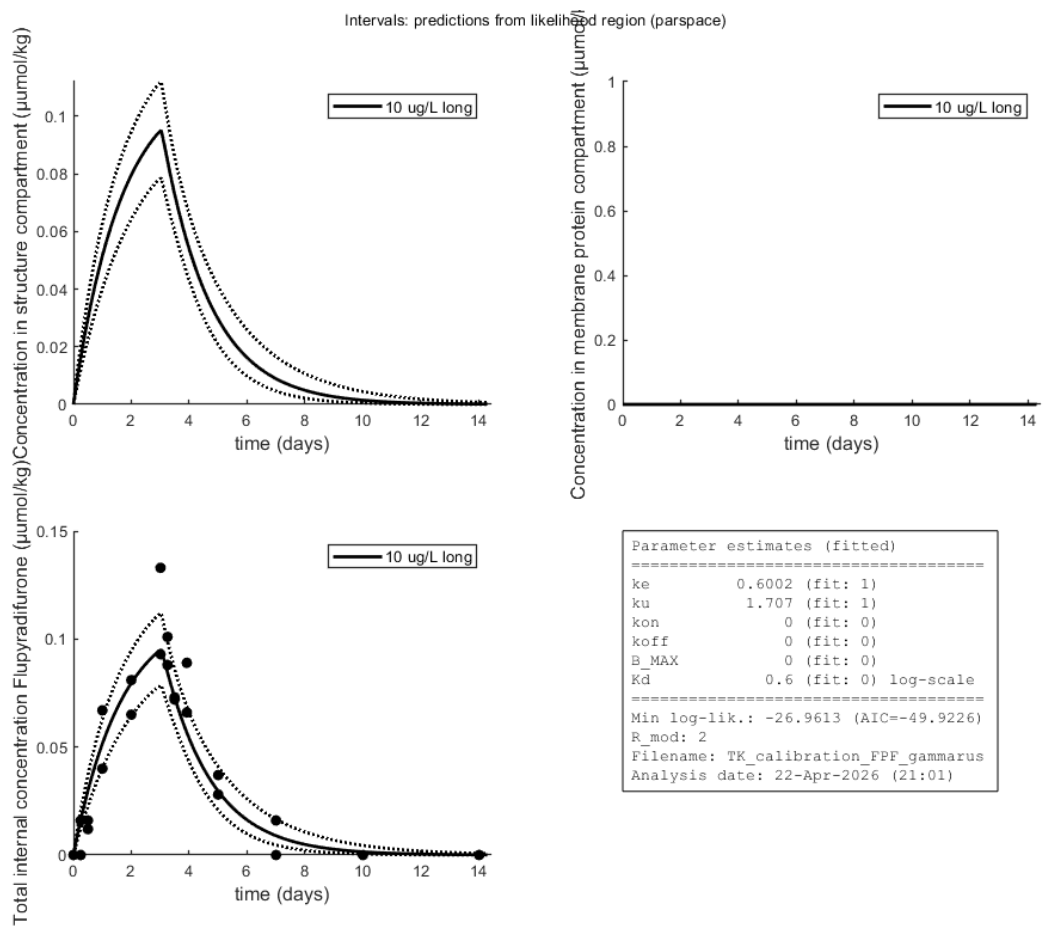

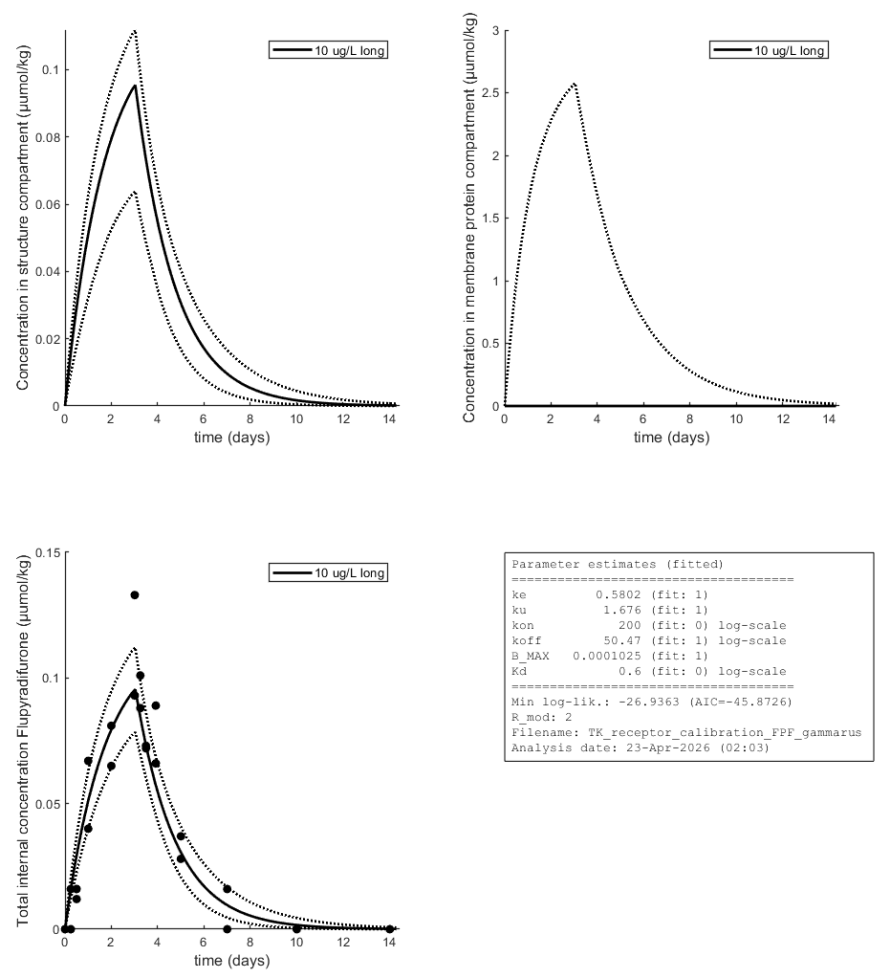

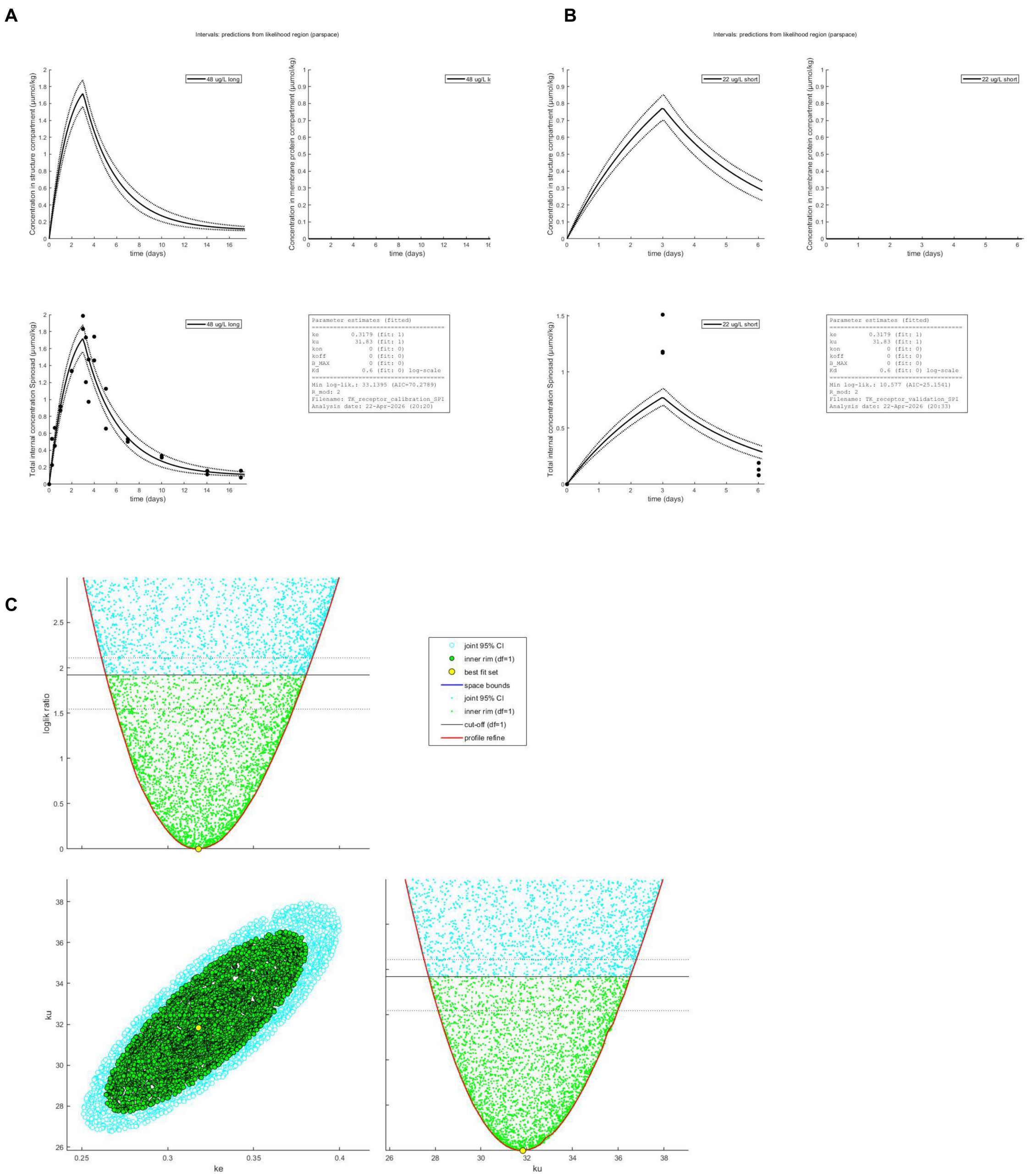

Figure SI-A12: Plots of (A) the model calibration, (B) the model validation, and (C) the parameter space of the calibration set for *G. pulex* exposed to Spinosad with a one-compartment model.

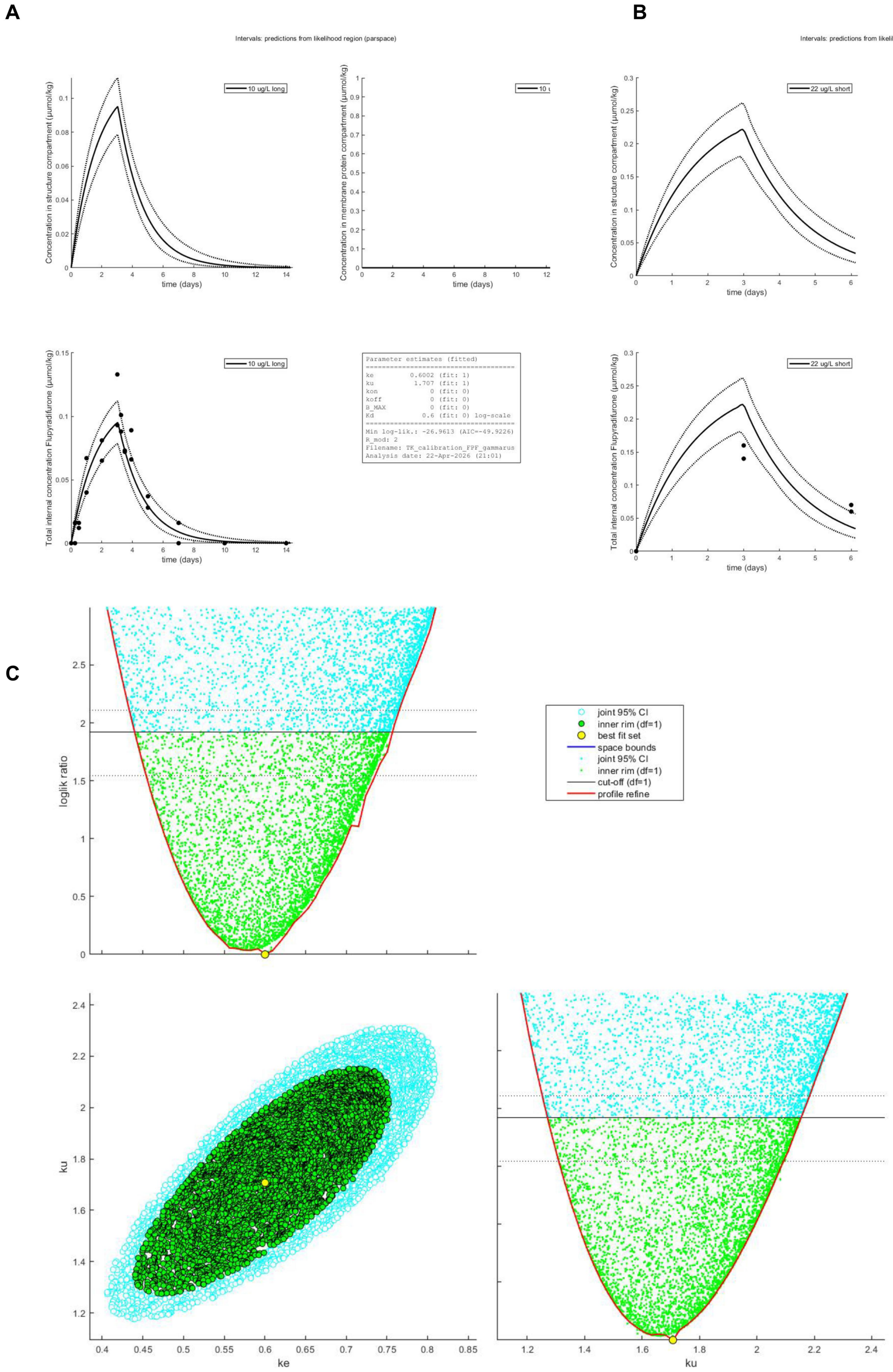

Figure SI-A13: Plots of (A) the model calibration, (B) the model validation, and (C) the parameter space of the calibration set for *G. pulex* exposed to Flupyradifurone with a one-compartment model.

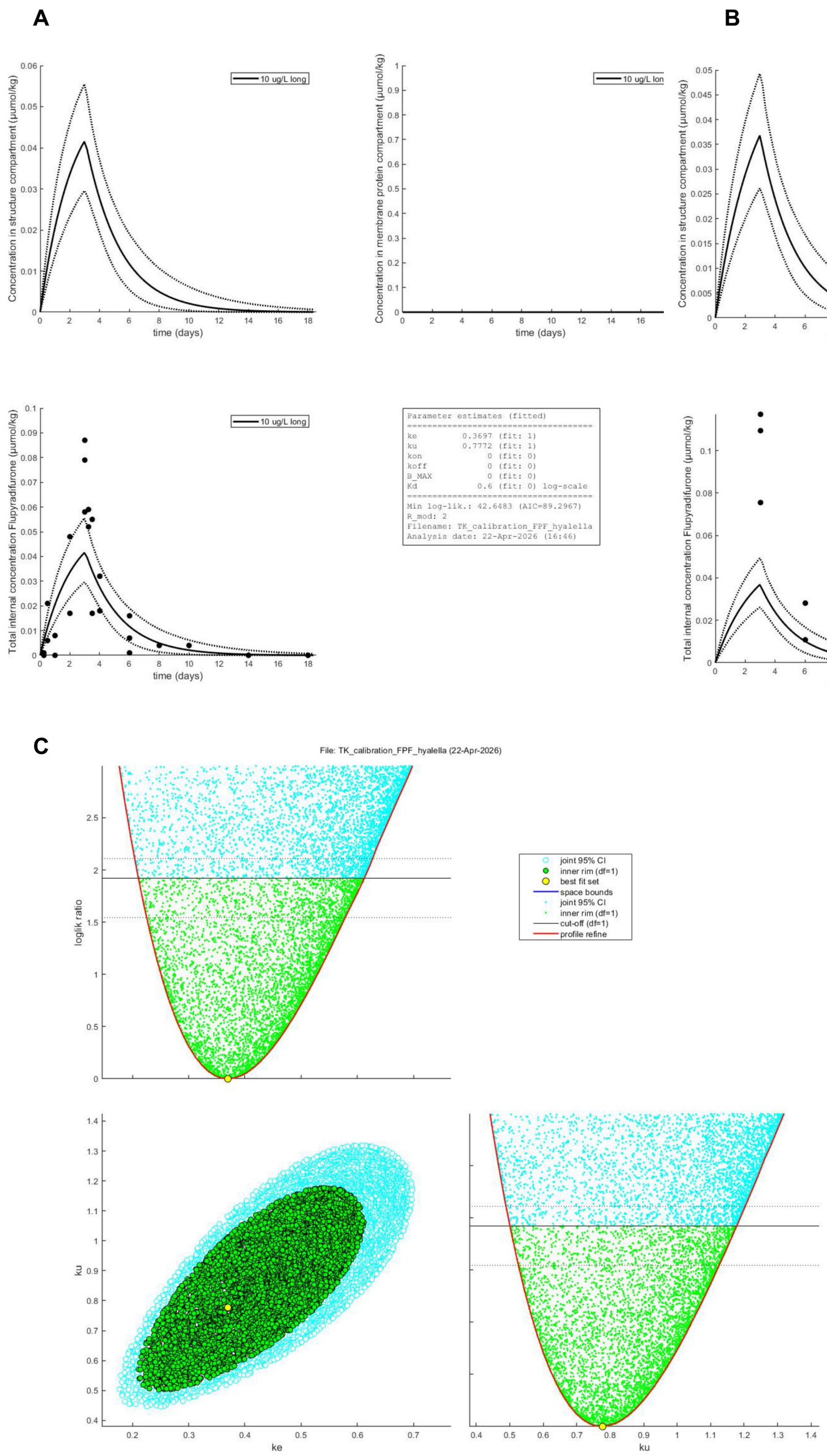

Figure SI-A14: Plots of (A) the model calibration, (B) the model validation, and (C) the parameter space of the calibration set for *H. azteca* exposed to Flupyradifurone with a one-compartment model.

SI-A-S5.5 Model evaluation

Based on the performance metrics compiled in Table SI-A14, the models generally show strong calibration, with calibration R^2 values consistently ranging from 0.89 to 0.99.
However, independent validation reveals the tendency toward systematic underprediction, as indicated by negative relative biases (%Bias) for all evaluated validation sets.
The model validation performance varies substantially across species and compounds, with the two-compartment models generally achieving higher concordance and explanatory power than the one-compartment models.

FPF modeling for *H. azteca* fails validation, evidenced by a negative R^2 (-0.21), an extreme CVRMSE (79.68%), and a severe underprediction bias (-60.57%). Looking at Figure SI-A14, both datasets share a narrow range of exposure concentrations, with biological variability of data points leading to the poor fit with overlapping confidence intervals.

Table SI-A14: Validation and model performance metrics (Root Mean Square Error (RMSE), Coefficient of Variation of RMSE (CVRMSE), Concordance Corr. Coeff. (CCC), and Relative Bias (%Bias)) alongside R^2 values for compounds and species.*Data and model fit published by Raths et al.[5].

**Compounds**	**Species**	R^2		**RMSE**	**CVRMSE**	**CCC**	**%Bias**
		**Calibration**	**Validation**	(μmol kg^{-1})	(%)		(%)
**Two-compartment model**							
THI	*G. pulex*	0.99*	0.99	0.03	10	0.96	-6
	H. azteca	0.93	0.87	0.29	35	0.55	-29
IMI	*G. pulex*	0.96	0.95	0.08	21	0.82	-20
	H. azteca	0.89	0.91	0.21	40	0.47	-36
ACE	*G. pulex*	0.94	0.99	0.05	24	0.66	-23
	H. azteca	0.97	0.98	0.02	11	0.96	-11
**One-compartment model**							
FPF	*G. pulex*	0.89	0.86	0.06	39	0.69	-37
	H. azteca	0.71	-0.21	0.01	80	0.67	-61
SPI	*G. pulex*	0.94	0.82	0.12	22	0.84	-21

SI-A-S5.6 Temperature considerations

Temperature corrections were performed using an unified Arrhenius Temperature of (T_A8050 K), which represents the mean of estimated values for uptake and elimination of THI in *G. pulex* (7800 K k_u; 8300 K for k_e)[4]. These values fall within the range of previously estimated values for a variety of polar organic contaminants (i.e., 7400 ± 2100 K *G. pulex*; 7900 ± 3100 K *H. azteca*) and closely match the measured physiological T_A for *H. azteca* (8000 ± 1600 K).[6]
Corrections were done for 15.0 ± 0.5 °C ($T_1 = 288.15 \pm 0.5$ K; $1/T \approx 0.00347$ K^{-1}) and 21 ± 1 °C ($T_2 = 294.15 \pm 1$ K; $1/T \approx 0.00340$ K^{-1}).
To normalize the toxicokinetic parameters, the experimentally derived rate constants (k) measured at the initial assay temperature (T_1) were adjusted to the target reference temperature (T_2) using the Arrhenius equation (3). Specifically, this equation was applied to scale the *G. pulex* data from 15 °C to 21 °C and the *H. azteca* data from from 21 °C to 15 °C.
The maximum binding capacity (B_{max}) was not temperature-corrected because, although the time to reach B_{max} varies with the temperature gradient, B_{max} itself is independent of temperature.[4]

$$\ln(k) = -T_A \left(\frac{1}{T}\right) + \ln(A) \tag{3}$$

Table SI-A15: Toxicokinetic parameters (k_u [L kg^{-1} d^{-1}] and k_e [d^{-1}]) for two- and one-compartment models across compounds, standardized at 15 °C and 21 °C using $T_A = 8050$ K).

		At 15 °C		At 21 °C	
**Compound**	**Species**	k_u	k_e	k_u	k_e
**Two-compartment model**					
THI	*G. pulex*	10.1 (8.1–13.0)	5.0 (4.0–6.4)	17.9 (14.3–22.9)	8.8 (7.1–11.3)
	H. azteca	5.5 (4.8–6.4)	1.0 (0.5–2.9)	9.7 (8.4–11.3)	1.7 (0.8–5.2)
IMI	*G. pulex*	3.0 (2.6–3.6)	1.7 (1.2–2.5)	5.3 (4.6–6.4)	3.0 (2.1–4.4)
	H. azteca	2.3 (1.9–2.7)	0.3 (0.1–1.0)	4.0 (3.3–4.8)	0.6 (0.2–1.8)
ACE	*G. pulex*	4.3 (3.3–6.0)	5.0 (3.0–10.8)	7.6 (5.8–10.6)	8.8 (5.3–19.1)
	H. azteca	2.8 (2.6–3.0)	0.1 (0.1–0.2)	4.9 (4.5–5.3)	0.2 (0.1–0.4)
**One-compartment model**					
FPF	*G. pulex*	1.7 (1.3–2.2)	0.6 (0.4–0.8)	3.0 (2.3–3.9)	1.1 (0.7–1.4)
	H. azteca	0.5 (0.3–0.7)	0.2 (0.1–0.3)	0.8 (0.5–1.2)	0.4 (0.2–0.6)
SPI	*G. pulex*	31.8 (27.7–36.5)	0.3 (0.3–0.4)	56.2 (49.0–64.5)	0.5 (0.5–0.7)

SI-A-S6 Supplementary results

SI-A-S6.1 Dissection

While literature reports the hemolymph volume of *G. pulex* to be $26 \pm 5\,\%$[16], our observations showed variable values, with a range of 0.6–$36.3\,\%$ relative to the average weight of the used organisms ($n = 12$). Because the total volume was not explicitly determined for each individual, concentrations are depicted without estimating the total content, also as quantification indicates losses during the dissection process (Figure SI-A15).

For IMI-ole, high concentrations were formed in the intestinal tissue during uptake (0.06 μmol kg^{-1}) but were depurated rapidly by $97\,\%$ in the elimination phase (< 0.01 μmol kg^{-1}), while the presence in the cephalon and carcass remained at similar levels during both the uptake and elimination phases, mirroring the total body burden and the persistence of parent IMI in these tissues (Figure SI-A15).

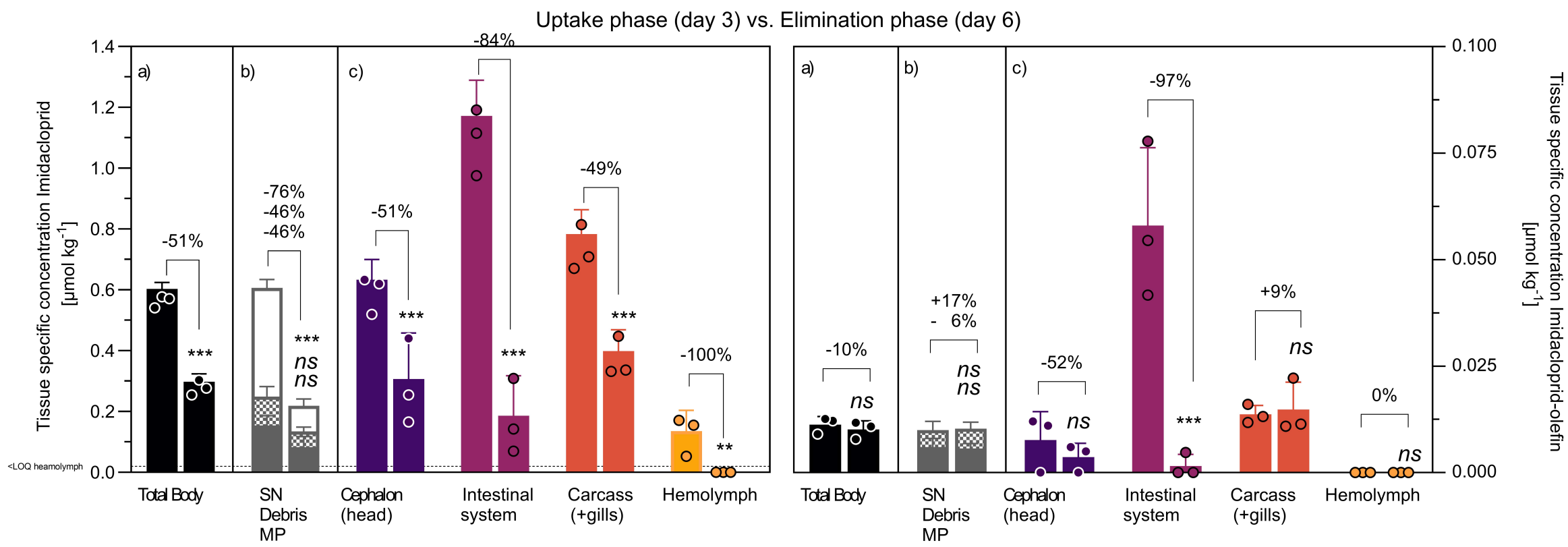

Figure SI-A15: Comparison of spatial distribution of imidacloprid concentrations (left y-axis) and its transformation product imidacloprid-olefin (right y-axis) in the uptake (left bar) and elimination (right bar) phase for a) the total body concentration (= tissue from whole body homogenate extracts), b) the in vivo assay (fractionated in supernatant (SN), debris and membrane proteins (MP), and c) in the dissected compartments. Data are presented as mean ± SD. Datasets a–c represent independent pool replicates, containing four animals per replicate for datasets a) and b), and six animals per replicate for dataset c). Please note that different y-axis scales have been used. The percentage (%) reduction from the elimination phase relative to the uptake phase is indicated above the elimination phase bar.

SI-A-S6.2 Long-term elimination experiment (60 days)

Data on a supplementary kinetic experiment performed in May to July 2026 where *G. pulex* were exposed to $10\ \mu$g L^{-1} thiacloprid for 1 day and left for elimination over 60 days.
Experimental conditions were 16℃ and mortality was 24% until day 31 and 44% until day 61.

Table SI-A16: Medium concentrations for THI during the uptake and elimination phase.

**Uptake duration**	**Uptake conc. [μg L^{-1}]**		**Elimination duration**	**Elimination conc. [μg L^{-1}]**	
	**Start**	**End**		**Start**	**End**
Day 0 to 1	9.3	10.0	Day 1 to 7	0.0	0.3
			Day 8 to 31	0.0	0.0
			Day 31 to 61	0.0	0.0

Table SI-A17: Tissue concentrations for THI over the experimental duration.

	**Tissue concentration [μg kg^{-1}]**					**Tissue concentration [μmol kg^{-1}]**				
**Time [d]**	**Replicates 1–5**					**Replicates 1–5**				
0	0.0	0.1	0.0	–	–	0.00	0.00	0.00	–	–
1	72.4	58.7	55.1	59.7	65.0	0.29	0.23	0.22	0.24	0.26
8	46.4	43.8	50.5	58.1	41.8	0.18	0.17	0.20	0.23	0.17
31	44.1	37.4	48.0	41.1	51.1	0.17	0.15	0.19	0.16	0.20
62	56.1	32.3	–	–	–	0.22	0.13	–	–	–

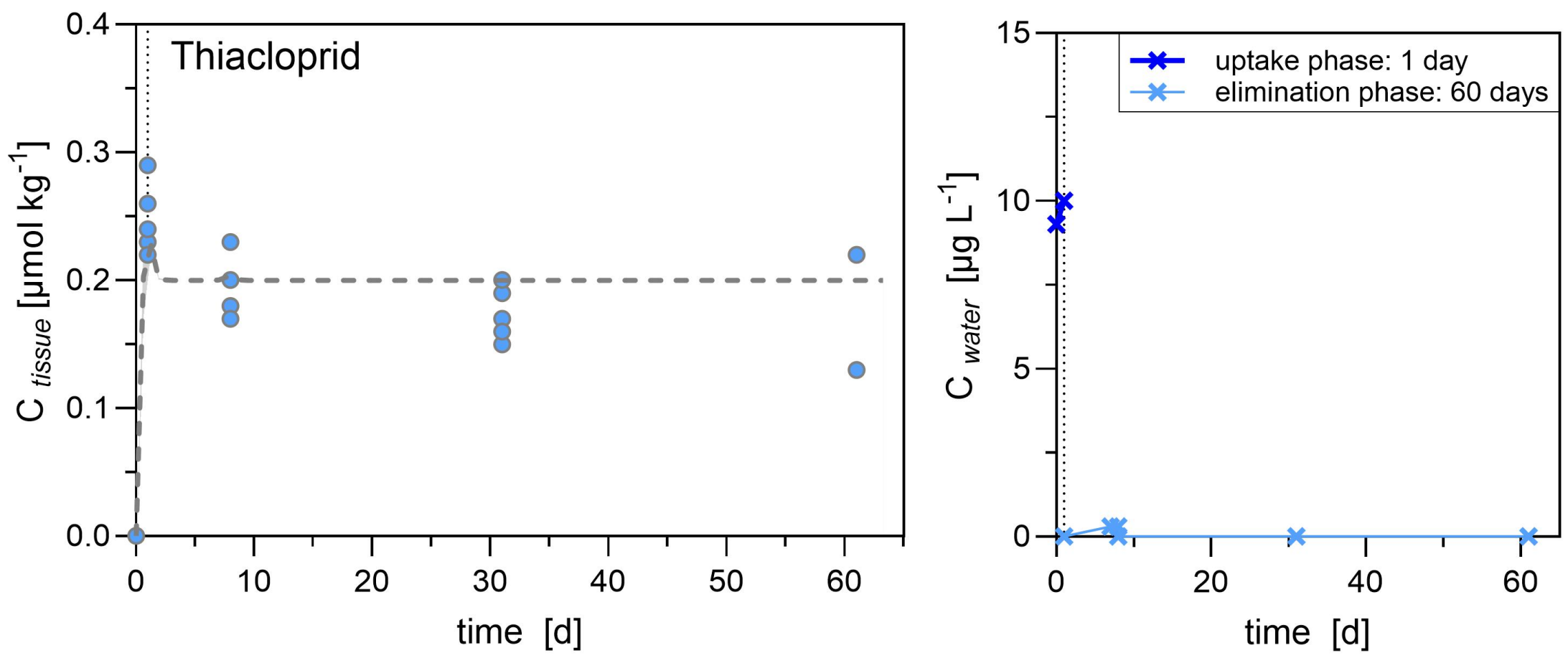

Figure SI-A16: Medium and Tissue concentration of *G. pulex* exposed to Thiacloprid for 1 day (uptake) and 60 days of elimination. Circles represent measured data points (n=5, except at 60 days, n=2 due to mortality) and the gray dashed line indicates the two-compartment model fit. The vertical dashed line indicates the change from the uptake to the elimination phase.

SI-A-S6.3 Bioconcentration factor (BCF)

The $\log K_{OW}$ values followed the order SPI (>4), THI (1.26) $>$ ACE (0.80) $>$ IMI (0.57) and FPF (0.08). Table SI-A18 compares different bioconcentration factors (BCF) that were determined.

The standard BCF ($BCF_{SS,\ total}$) was calculated as the ratio of the average concentration in the organism to the average concentration in the water at the end of the uptake phase (eq.4). Due to a saturation of the second, elimination-resistant MP compartment, values show concentration dependence of the whole-body bioconcentration.

$$BCF_{SS,\ total} = \frac{C_{tissue}}{C_{water}} \tag{4}$$

In addition, BCFs were derived from the applied kinetic models (BCF_{kin}) as the ratio of k_u and k_e. For the neonicotinoids (i.e., THI, IMI, and ACE) the BCF_{kin} was derived from the two-compartment model, and for FPF and SPI from a one-compartment model:

$$BCF_{kin} = \frac{k_u}{k_e} \tag{5}$$

Both standard BCFs do not consider the elimination-resistant fraction. Thus, in addition, for the neonicotinoids we calculated the BCF ($BCF_{SS,\ structure}$) corrected by the elimination-resistant fraction in the elimination phase[5]. Thus, the amount bound to the receptor at the end of the elimination phase is subtracted from the concentration at the end of the uptake phase:

$$BCF_{SS,\ structure} = \frac{C_{tissue,\ uptake} - C_{tissue,\ elimination}}{C_{water}} \tag{6}$$

To evaluate the bioaccumulation potential of the elimination-resistant fraction we calculated the critical water concentration (ng L^{-1} range) below which the regulatory BCF threshold of 2000 (OECD 321) would be exceeded applying equation 7. Where FMS describes the factor for the proportional correction of the membrane protein volume (i.e., FMS = 0.008 *G. pulex* and 0.009 *H. azteca*). The BCF would continually increase at lower, field-relevant concentrations.

$$C_{water,\ crit} = \frac{B_{max} \times FMS}{BCF_{threshold}} \tag{7}$$

Once the BCF_{kin} is normalized to the lipid and protein content (Table SI-A19), the normalization narrows the quantitative difference in bioaccumulation between species without altering that *H. azteca* exhibits higher BCFs than *G. pulex* neonicotinoids but lower for the butenolid.

Table SI-A18: Determined BCFs (Lkg^{-1}) for the compounds thiacloprid (THI), imidacloprid (IMI), acetamiprid (ACE), flupyradifurone (FPF) and spinosad (SPI) for *G. pulex* and *H. azteca*, respectively. Values corresponding to data published by Raths et al.[5].

**Compounds**	**Species**	**BCF [L kg^{-1}]**			**Critical C_{water} [ng L^{-1}]**
		steady-state (Eq.4)	*kinetic* (Eq.5)	*2-compartment* (Eq.6)	$BCF_{>2000}$ (Eq.7)
THI	*G. pulex**	$1.9 \pm 0.2 - 109 \pm 14$	2.0 (1.3–3.3)	2.5 ± 0.8	30
	G. pulex	4.1 ± 0.5		2.5	25 (23–30)
	H. azteca	$14.8 \pm 2 - 18.5 \pm 0.7$	5.6 (1.6–12.8)	5.4 ± 1.6	72 (63–81)
IMI	*G. pulex*	$2.6 \pm 0.2 - 7.2 \pm 0.0$	1.7 (0.3–1.0)	1.8 ± 0.4	31 (28–33)
	H. azteca	$3.1 \pm 0.2 - 13.3 \pm 5.8$	6.8 (1.9–18.1)	3.4 ± 3.8	38 (25–48)
ACE	*G. pulex*	$1.9 \pm 0.3 - 8.5 \pm 0.6$	0.9 (0.3–2.0)	1.8 ± 0.7	16 (14–18)
	H. azteca	$2.0 \pm 0.1 - 21.6 \pm 0.7$	21.8 (11.6–44.3)	2.8 ± 3.1	14 (10–18)
FPF	*G. pulex*	$1.6 \pm 0.2 - 2.8 \pm 0.7$	2.8 (1.7–4.9)		
	H. azteca	$2.4 \pm 0.5 - 3.7 \pm 0.8$	2.1 (0.8–5.6)		
SPI	*G. pulex*	$29 \pm 2 - 47 \pm 7$	100 (73–138)		

Table SI-A19: Normalized bioconcentration factors for *G. pulex* (Lipid: 1.7%, Protein: 4.4%) and *H. azteca* (Lipid: 3.3%, Protein: 6.2%). Protein-normalized BCFs are calculated using the actual measured protein fraction.

**Compounds**	**Species**	$\mathbf{BCF}_{\text{kin}}$	$\mathbf{BCF}_{\text{lipid (5\%)}}$	$\mathbf{BCF}_{\text{protein}}\left(\frac{\mathbf{BCF}_{\text{kin}}}{f_{\text{protein}}}\right)$
THI	*G. pulex*	2.0 (1.3–3.3)	5.9 (3.8–9.7)	46 (30–75)
	H. azteca	5.6 (1.6–12.8)	8.5 (2.4–19.4)	90 (26–207)
IMI	*G. pulex*	1.7 (0.3–1.0)	5.0 (0.9–2.9)	39 (7–23)
	H. azteca	6.8 (1.9–18.1)	10.3 (2.9–27.4)	110 (31–292)
ACE	*G. pulex*	0.9 (0.3–2.0)	2.6 (0.9–5.9)	21 (7–46)
	H. azteca	21.8 (11.6–44.3)	33.0 (18–67)	352 (187–715)
FPF	*G. pulex*	2.8 (1.7–4.9)	8.2 (5.0–14.4)	64 (39–111)
	H. azteca	2.1 (0.8–5.6)	3.2 (1.2–8.5)	34 (13–90)
SPI	*G. pulex*	100 (73–138)	294 (215–406)	2273 (1659–3136)

SI-A-S6.4 *In vivo* binding

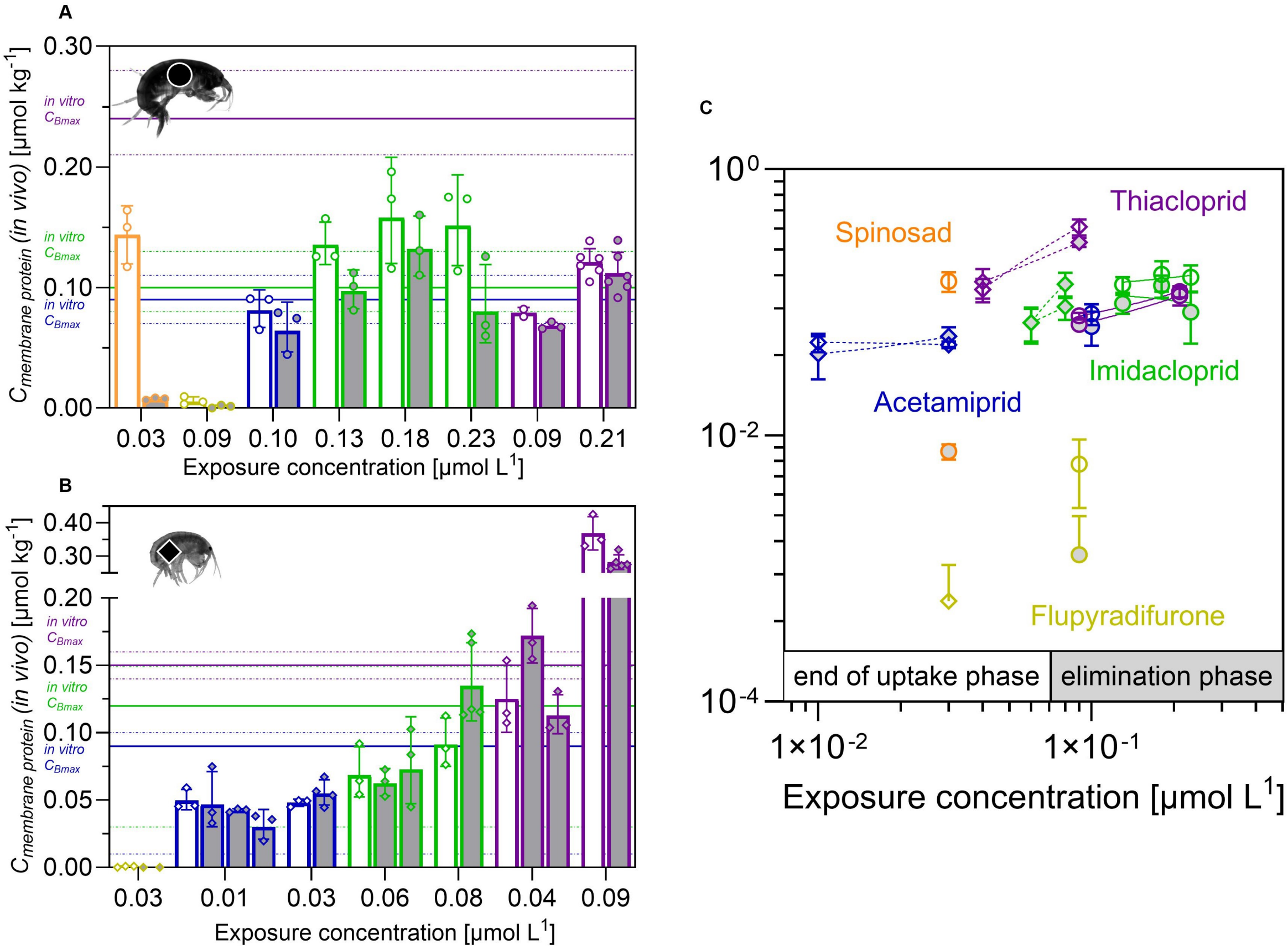

Figure SI-A17: Concentrations [μmol kg^{-1}] in the membrane-bound fraction relative to the exposure concentration from *in vivo* binding for A) *G. pulex*, B) *H. azteca* and C) both combined. Concentrations correspond to the dataset presented in the inset graphs in the manuscript in Figure 3 and Figure 4 (*G. pulex*: circles; *H. azteca*: diamonds).
In panel A and B, bars indicate mean measured values $\pm$ SD (uptake phase: white, elimination phase: grey), while horizontal solid and dashed lines depict the in vitro extrapolated $C_{B\max}$ values (SI-A30) and their corresponding confidence intervals. In panel C, solid lines (*G. pulex*) and dashed lines (*H. azteca*) represent separate simple linear regressions calculated for the uptake (white bars/symbols) and elimination (grey bars/symbols) phases.
Colors represent the different compounds for acetamiprid in blue, thiacloprid and imidacloprid in green, flupyradifurone in yellow, and spinosad in orange.

SI-A-S6.5 Biotransformation

While ACE-N-desmethyl was only detected in the uptake phase, IMI-ole remained detectable during the elimination phase (Figure SI-A19). Quantified concentrations remain within the standard deviations of the parent compound, having a minor contribution to the total body burden. Unfortunately, the data lacks the sensitivity to establish a definitive mass balance to distinguish between continued metabolism of IMI and other factors (e.g., affinity). Indeed, for *G. pulex*, two time points are available, preventing strong conclusions. For *H. azteca*, the absolute values of IMI-ole increase first while the parent IMI undergoes rapid depuration (compared to Figure 3, IMI). Despite this initial rise in the first 7 days of elimination and subsequent decrease in absolute amounts during the last four elimination days, the relative contribution remains the same, ranging around 6%. This stable relative proportion is comparable to the elimination-resistant fraction of IMI, and the interplay with growth as an influencing factor is difficult to assess. Additionally, Figure SI-A18 depicts higher absolute values for the experiment at the lower concentration than for the higher one„ making definitive conclusions difficult. This variability highlights a key area of great interest for future research.

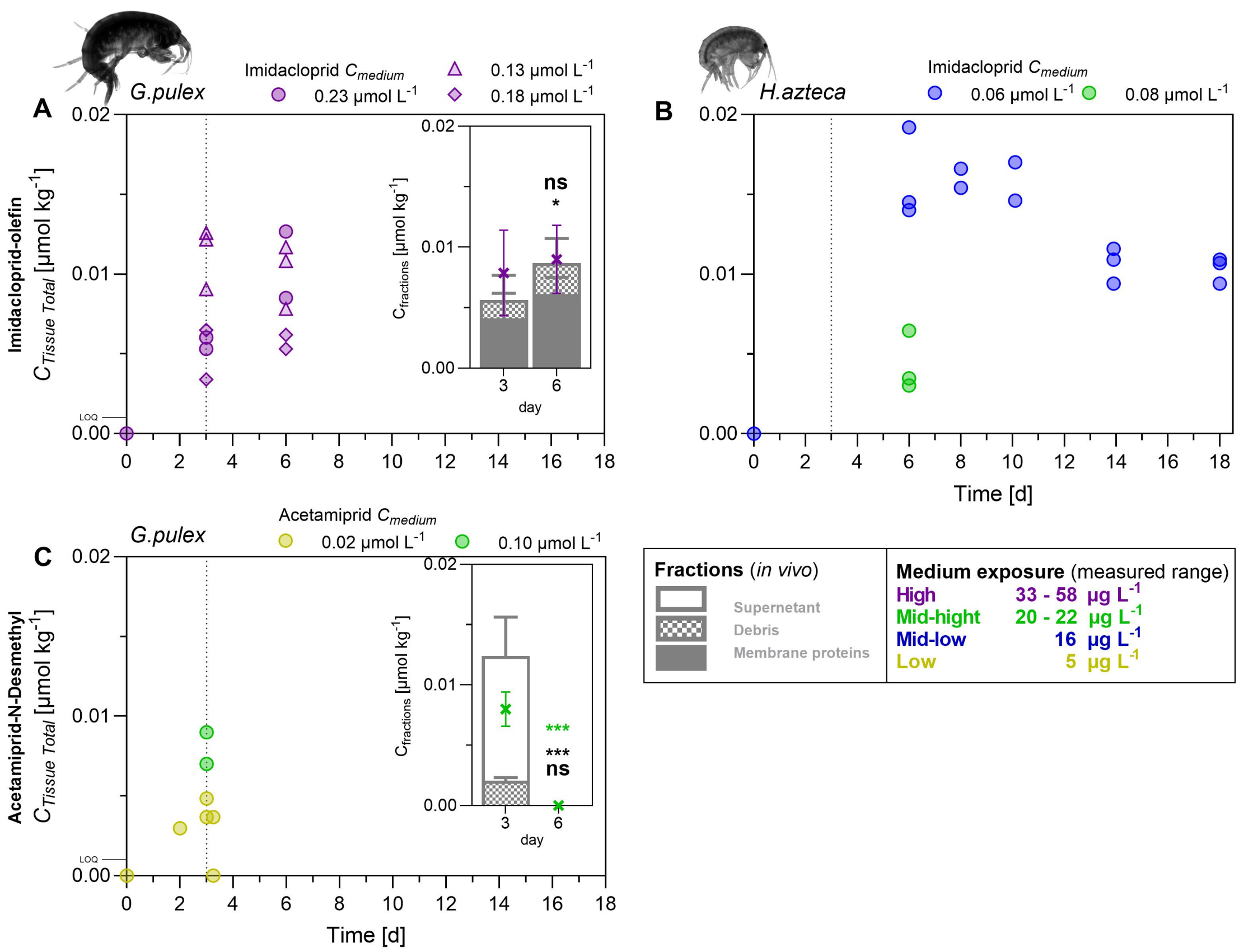

Figure SI-A18: Imidacloprid-olefin (A, B) and Acetamiprid *N*-Desmethyl (C) and concentrations [μmol kg^{-1}] recovered in total tissue and different fractions of the *in vivo* binding assay for *G. pulex* (A, C) and *H. azteca* (B), respectively. The recovery of the different fractions equals the fraction from total tissue analysis, yet with low precision, as quantification was close to the LOQ (debris) and <LOQ (SN).

SI-A-S6.6 *In vitro* binding

The sensitivity of the *in vitro* assay does not allow determination of a reliable equilibrium dissociation constant (K_d) due to rapid binding kinetics and a high ligand-to-membrane protein ratio. Lower exposure concentrations to balance this ratio were precluded by the analytical sensitivity limits.

We further noted that extrapolating *in vitro* receptor density ($B_{\max}$) to the whole organism ($C_{B\max}$) introduces significant uncertainty, as the calculation of $C_{B\max}$ requires correcting for both recovery and total MP content (*FMS*):

$$C_{B\max} = \frac{B_{\max} \cdot FMS}{REC} \tag{8}$$

Even minor variations in these parameters can dramatically shift the resulting scale. E.g., in this study, MP recovery fluctuated between 21%, 25%, and 50% across the assays (Table SI-A7), while the MP content ranged between 0.7–0.9% depending on the species (Table SI-A2). For example, the $C_{B\max}$ calculated for THI *H. azteca* would result in 0.23 μmol kg^{-1} for the first assay and 0.11 μmol kg^{-1} in the second due to variations in the recovery of 25% and 50%, respectively. This discrepancy highlights the difficulty of accurately extrapolating *in vitro* to *in vivo* results.

Table SI-A20: Receptor binding parameters estimated with the one-site, specific binding model. Parameters are presented with the corresponding 95% CI and R^2 derived from lines of best fit shown in Figure 3 in the manuscript. $C_{B\max}$ = maximal nAChR bound extrapolated to the whole body of *G. pulex* and *H. azteca*, respectively (eq. 8).

**Compound**	R^2	$B_{\max}$ [pmol mg$_{\text{MP}}^{-1}$]		K_d [nM]		$C_{B\max}$ [μmol kg^{-1}]	
		Best fit	95% CI	Best fit	95% CI	Best fit	95% CI
G. pulex							
THI*	0.59	5.9	5.2 – 6.9	0.73	0.22 – 1.7	0.24	0.21 – 0.28
IMI	0.71	2.5	2.1 – 3.4	0.99	−0.11 – 3.6	0.10	0.08 – 0.13
IMI-olefin	0.89	0.6	0.4 – 0.9	0.43	−0.22 – 2.8	0.02	0.01 – 0.04
ACE	0.69	2.4	1.9 – 2.9	0.15	−0.45 – 1.2	0.09	0.07 – 0.11
FPF	0.09	0.4	−0.55 – inf.	–	–	0.02	0.00 – 0.02
H. azteca							
THI	0.95	6.4	6.0 – 6.8	0.12	−0.06 – 0.34	0.15	0.14 – 0.16
IMI	0.71	5.1	4.0 – 6.3	0.55	0.05 – 1.30	0.12	0.03 – 0.15
IMI-olefin	0.99	4.9	4.4 – 5.4	0.43	0.25 – 0.67	0.11	0.01 – 0.13
ACE	0.63	3.9	3.3 – 4.5	0.66	0.13 – 1.40	0.09	0.01 – 0.10
FPF	n.d.						

*Best fit combines the data obtained in this study together with the dataset published by Raths et al.[5] where the $C_{B\max}$ for THI was calculated as 0.24 μmol kg^{-1} based on a 24% recovery rate and a 1% MP content.

Additional data from *G. pulex* (Netherlands strain, (Table SI-A7)) confirmed that while the absolute $B_{\max}$ may vary between populations (compared to the Switzerland strain), the hierarchical affinity of the compounds (THI $>$ IMI $>$ ACE $>$ FPF) remains consistent (Figure SI-A19). To explain these observed variations, we compared the toxicokinetic data obtained in our study with the results published by Huang et al.[10,17] for *G. pulex* exposed to IMI and FPF. Both datasets are largely comparable (Figure SI-A20) and do not explain the observed difference (Figure SI-A19). Thus, we hypothesized that the transport of the organisms may have affected the *in vivo* binding assay (e.g., potentially altering protein stability).
Toxicokinetics showed faster depuration rates (FPF $k_e = 0.6$ d^{-1}, IMI $k_e = 1.7$ d^{-1}) than previously reported (FPF $k_e = 0.21$ d^{-1}, IMI $k_e = 0.12$ d^{-1})[10,17], while the uptake rates were slower. The faster depuration for IMI can be explained by the application of the two-compartment model. However, we questioned the difference observed for FPF. We hypothesized that the procedural difference of food provided in this study during the elimination phase accelerated the elimination processes and outweighed any potential temperature differences between the experiments (the Wageningen *G. pulex* population was maintained at $18\,^{\circ}$C, whereas ours was kept at $15\,^{\circ}$C).

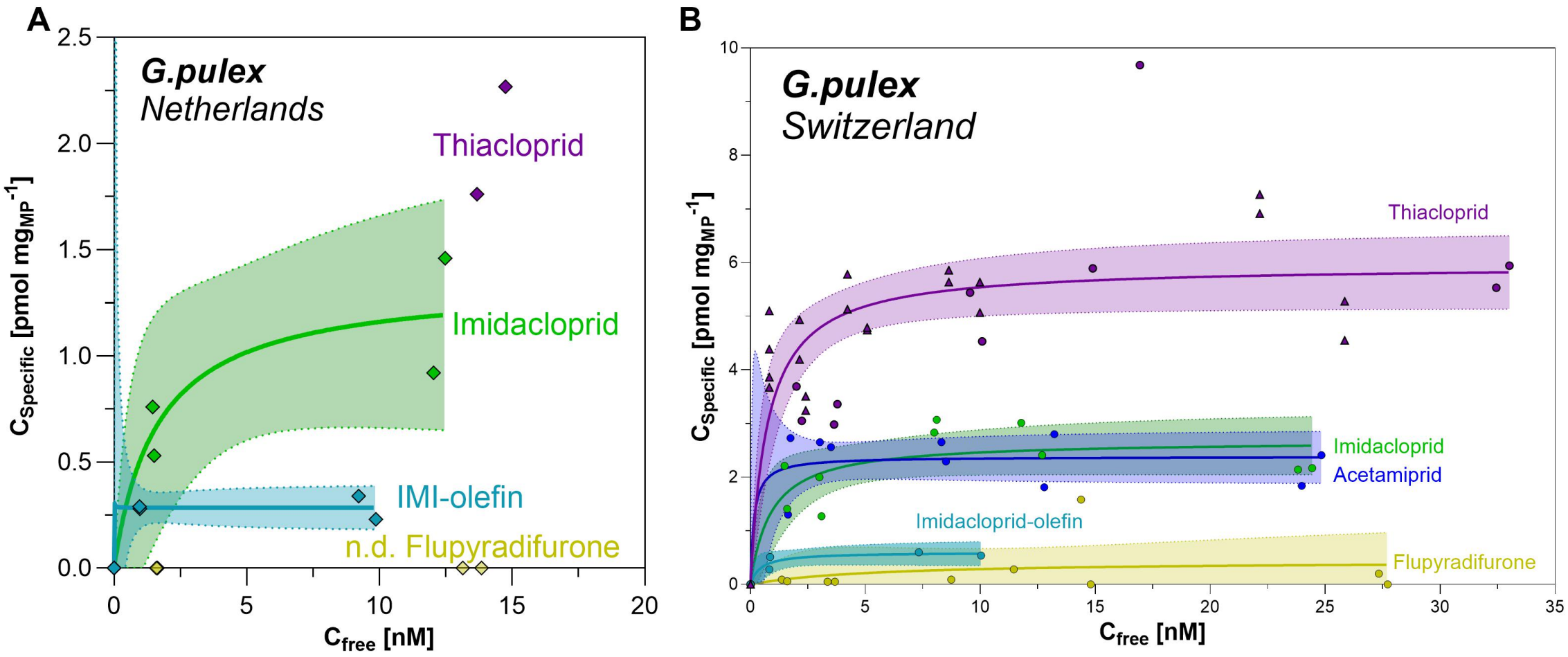

Figure SI-A19: Saturation curve of the *in vitro* receptor binding assay using membrane proteins from *G. pulex* from A) the Netherlands and B) from Switzerland (Table SI-A7). The non-linear regression of the one-site, specific-binding model and the 95% CIs are presented as a line and shaded area, respectively. Symbols represent measured data, while the colors represent the different compounds for THI = thiacloprid in violet, IMI = imidacloprid in green, IMI-o = imidacloprid-olefin in turquoise, and FPF = flupyradifurone in yellow.

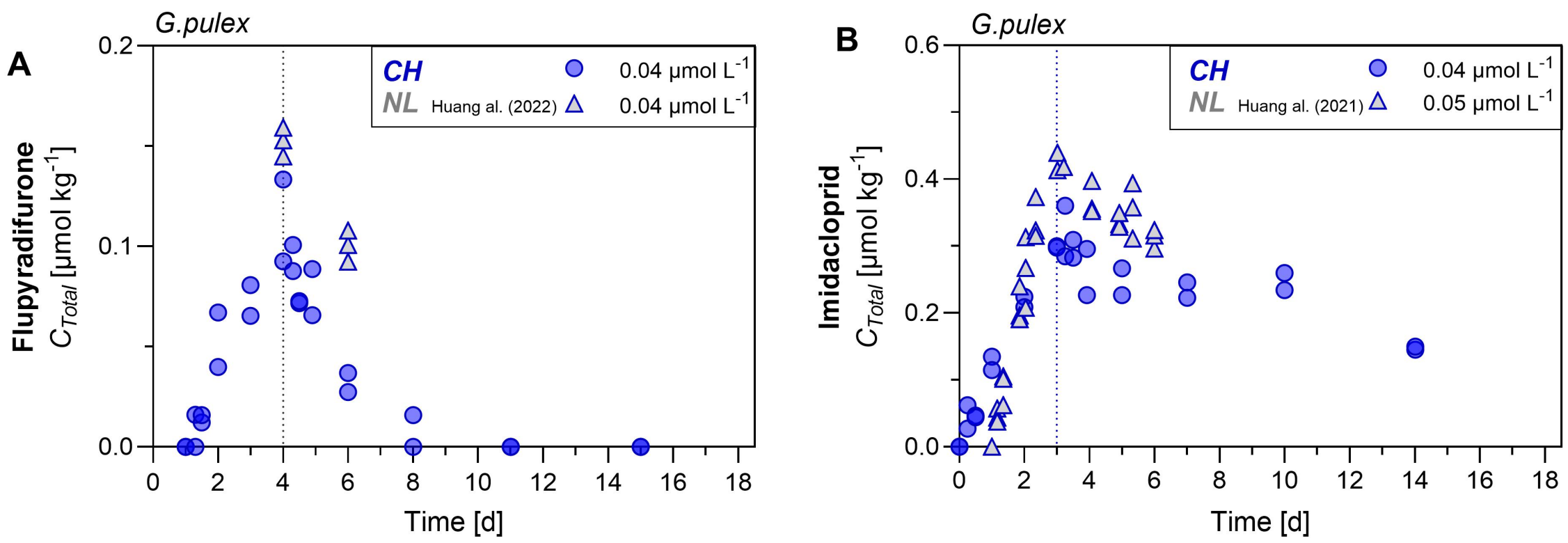

Figure SI-A20: Comparison of the tissue concentrations [μmol kg^{-1}] for A) Flupyradifurone and B) Imidacloprid recovered from the experiment conducted with *G. pulex* in Switzerland (CH, blue dots) in this study and *G. pulex* in the Netherlands (NL, gray triangles) initially published by Huang et al.[10,17].

References

1. Agriculture & (AERU), E. R. U. *Pesticide Properties Database (PPDB)* 2026.
2. Agriculture & (AERU), E. R. U. *Biopesticides Database (BPDB)* 2026.
3. Glaberman, S., White, K., Steeger, T., Carleton, J. & Winfield, S. *Environmental Fate and Ecological Risk Assessment for Foliar, Soil Drench, and Seed Treatment Uses of the New Insecticide Flupyradifurone (BYI 02960) Flupyradifurone* tech. rep. (Environmental Protection Agency (EPA), 2014).
4. Raths, J., Hermann, M., von Au, C. & Hollender, J. A moment of heat, a lifetime exposure? Integrating effects of temperature and heatwaves into toxicokinetic modelling of a neonicotinoid in aquatic invertebrates. *Environment International* **214,** 110424. doi:10.1016/J.ENVINT.2026.110424 (2026).
5. Raths, J., Schinz, L., Mangold-Döring, A. & Hollender, J. Elimination Resistance: Characterizing Multi-compartment Toxicokinetics of the Neonicotinoid Thiacloprid in the Amphipod Gammarus pulex Using Bioconcentration and Receptor-Binding Assays. *Environmental Science and Technology* **57,** 8890–8901. doi:10.1021/acs.est.3c01891 (2023).
6. Raths, J., Švara, V., Lauper, B., Fu, Q. & Hollender, J. Speed it up: How temperature drives toxicokinetics of organic contaminants in freshwater amphipods. *Global Change Biology* **29,** 1390–1406. doi:10.1111/gcb.16542 (2023).
7. Sroda, S. & Cossu-Leguille, C. Seasonal variability of antioxidant biomarkers and energy reserves in the freshwater gammarid Gammarus roeseli. *Chemosphere* **83,** 538–544. doi:10.1016/J.CHEMOSPHERE.2010.12.023 (2011).
8. Dalhoff, K., Gottardi, M., Rinnan, Å., Rasmussen, J. J. & Cedergreen, N. Seasonal sensitivity of Gammarus pulex towards the pyrethroid cypermethrin. *Chemosphere* **200,** 632–640. doi:10.1016/j.chemosphere.2018.02.153 (2018).
9. Beketov, M. A. & Liess, M. Potential of 11 pesticides to initiate downstream drift of stream macroinvertebrates. *Archives of Environmental Contamination and Toxicology* **55,** 247–253. doi:10.1007/s00244-007-9104-3 (2008).
10. Huang, A., Mangold-Döring, A., Focks, A., Zhang, C. & Van den Brink, P. J. Comparing the acute and chronic toxicity of flupyradifurone and imidacloprid to non-target aquatic arthropod species. *Ecotoxicology and Environmental Safety* **243,** 113977. doi:10.1016/j.ecoenv.2022.113977 (2022).
11. Beketov, M. A. & Liess, M. Acute and delayed effects of the neonicotinoid insecticide thiacloprid on seven freshwater arthropods. *Environmental Toxicology and Chemistry* **27,** 461–470. doi:10.1897/07-322R.1 (2008).
12. Raby, M. *et al.* Acute toxicity of 6 neonicotinoid insecticides to freshwater invertebrates; Acute toxicity of 6 neonicotinoid insecticides to freshwater invertebrates. *Environ Toxicol Chem* **37,** 1430–1445. doi:10.1002/etc.4088 (2018).
13. Bartlett, A. J. *et al.* Acute and chronic toxicity of neonicotinoid and butenolide insecticides to the freshwater amphipod, Hyalella azteca. *Ecotoxicology and Environmental Safety* **175,** 215–223. doi:10.1016/j.ecoenv.2019.03.038 (2019).
14. Jager, T. & Kooijman, S. A. Modeling receptor kinetics in the analysis of survival data for organophosphorus pesticides. *Environmental Science and Technology* **39,** 8307–8314. doi:10.1021/es050817y (2005).
15. Landrum, P. F., Lee, H. & L Y D Y, M. J. Annual Review TOXICOKINETICS IN AQUATIC SYSTEMS: MODEL COMPARISONS AND USE IN HAZARD ASSESSMENT. *Environmenlal Toxicology and Chemistry* **11,** 1709–1725. doi:10.1002/etc.5620111205 (1992).

16. Butterworth, P. E. *An estimation of haemolymph volume in gammarus pulex* tech. rep. (University of Cambridge, 1968), 1123–1125.
17. Huang, A., van den Brink, N. W., Buijse, L., Roessink, I. & van den Brink, P. J. The toxicity and toxicokinetics of imidacloprid and a bioactive metabolite to two aquatic arthropod species. *Aquatic Toxicology* **235,** 105837. doi:10.1016/j.aquatox.2021.105837 (2021).
